# Profile of the gut microbiota of Pacific white shrimp under industrial indoor farming system

**DOI:** 10.1007/s00253-024-13046-0

**Published:** 2024-02-20

**Authors:** Meng Li, Abdallah Ghonimy, Dai-Qiang Chen, Ji-Tao Li, Yu-Ying He, Laura Susana López Greco, Fernando Dyzenchauz, Zhi-Qiang Chang

**Affiliations:** 1 Key Laboratory of Sustainable Development of Marine Fisheries, Key Laboratory of Maricultural Organism Disease Control, Ministry of Agriculture and Rural Affairs, Yellow Sea Fisheries Research Institute, Chinese Academy of Fishery Sciences, Qingdao, 266071 People’s Republic of China; 2 College of Fisheries and Life Science, Shanghai Ocean University, Shanghai, 201306 People’s Republic of China; 3 Function Laboratory for Marine Fisheries Science and Food Production Processes, Laoshan Laboratory, Qingdao, 266071 People’s Republic of China; 4 Departamento de Biodiversidad y Biología Experimental, Laboratorio de Biología de la Reproducción y el Crecimiento de Crustáceos Decápodos, Universidad de Buenos Aires, CONICET, Instituto de Biodiversidad y Biología Experimental y Aplicada (IBBEA, UBA-CONICET), Facultad de Ciencias Exactas y Naturales, 1428EGA Buenos Aires, Argentina

**Keywords:** Gut microbiota, *Litopenaeus vannamei*, Indoor shrimp farming, Developmental stages, Environment

## Abstract

**Abstract:**

The gut microbial communities interact with the host immunity and physiological functions. In this study, we investigated the bacterial composition in *Litopenaeus vannamei* shrimp’s gut and rearing water under different host (developmental stage: juvenile and adult; health status: healthy and diseased) and environmental factors (temperature 25 °C and 28 °C; and light intensity: low and high). The PCoA analysis showed that all water samples were clustered together in a quarter, whereas the gut samples spread among three quarters. In terms of functional bacteria, gut samples of adult shrimp, healthy adult shrimp, adult shrimp raised at 28 °C, and juvenile shrimp under high light intensity exhibited a higher abundance of *Vibrionaceae* compared to each other opposite group. Gut samples of juvenile shrimp, infected adult shrimp, juvenile shrimp with low light intensity, and adult shrimp with a water temperature of 25 °C showed a higher abundance of *Pseudoaltromonadaceae* bacteria compared to each other opposite group. Gut samples of juvenile shrimp, healthy adult shrimp, adult shrimp raised at a water temperature of 28 °C, and juvenile shrimp with high light intensity showed the higher abundance of *Firmicutes*/*Bacteroidota* ratio compared to each other opposite group. Our results showed that *L. vannamei* juveniles are more sensitive to bacterial infections; besides, water temperature of 28 °C and high light intensity groups were both important conditions improving the shrimp gut bacterial composition under industrial indoor farming systems.

**Key points:**

*• Bacteria diversity was higher among shrimp intestinal microbiota compared to the rearing water*.

*• Shrimp juveniles are more sensitive to bacterial infection compared to adults*.

*• Water temperature of 28 °C and high light intensity are recommended conditions for white shrimp aquaculture*.

**Graphical Abstract:**

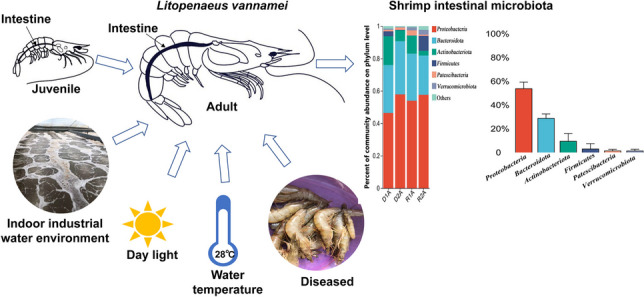

**Supplementary Information:**

The online version contains supplementary material available at 10.1007/s00253-024-13046-0.

## Introduction

Over the last two decades, the aquaculture sector has been increasingly recognized as the main driving force for food supply and an essential contribution to global food security. By now, global aquaculture production has reached a record of 122.6 million tons, of which 71% were from aquatic animals and worth 264.8 billion USD (FAO 2022). In fact, shrimp has historically been the major coastal cultured species and the most heavily traded aquatic commodity in the world, due to their high economical and nutritional values (Lebel et al. 2010; Fan et al. 2019). In 2020, shrimp was the main exported species accounting for 16.4% of the total aquatic products in value terms (FAO 2022), where China produced 20.6% of shrimp species in the coastal and inland production areas, and much of the shrimp are raised using outdoor earthen ponds. With rapid development of the shrimp aquaculture around the world, infectious diseases caused by various microorganisms (such as bacteria or fungi), viruses, protozoa, and a small amount of parasites have become the most common challenge in shrimp-farming industry and have resulted in a huge economic loss (Thitamadee et al. 2016; Xiong et al. 2016; Holt et al. 2021).

Recently, the FAO has proposed a vision of Blue Transformation, promoting innovative approaches that expand the contribution of aquatic foods to global food security, through affordable, highly nutritious, and healthy values. To achieve this goal, further farming technical innovations are indispensable (FAO 2022). Farming models and technologies have been developed along with social economy development in shrimp-producing countries, where several intensive shrimp-farming models with a high stocking density and productivity drive the rapid growth of shrimp aquaculture industry (Chang et al. 2020; Joffre et al. 2018; Jory 2018). In comparison with traditional outdoor earthen pond aquaculture, indoor farming systems open the opportunity for a constant year-round production in locations with seasonal temperature fluctuations (Martins et al. 2010; Ray et al. 2017). These farming models include a flow-through system (FTS) and a recirculating aquaculture system (RAS), which are both recognized as clean and bio-secure systems with low risk of disease infection, and exhibiting low chemicals and heavy metal accumulation (Poppick 2018; Naylor et al. 2021; Timmons et al. 2018).

In recent years, an industrial indoor shrimp-farming (IISF) model developed for aquaculture on the Pacific white shrimp *Litopenaeus vannamei* has gained much attention, being adopted widely in northern China, especially in coastal area of the Shandong province, due to their high production yield, over 10 kg/m^2^/cycle with a more stable water quality (Chang et al. 2020). There are mainly two types of IISF models in northern China, based on their roof thermal preservation property of farming facilities in winter. In one type, the roof is covered with a plastic sheet allowing light penetration and consequently increasing room temperature; it is a popular warming method in central and southern areas of China. In the other type, the roof is covered with a thick glass-wool acting as a thermal insulator and preventing the light penetration, being a popular warming method in the northern colder areas of China. FTS has been used in the majority of IISF to maintain the water quality, while RAS was adopted as well, but in fewer shrimp farms (Chang et al. 2020). However, with the increase of stocking density, the onset of disease under intensive shrimp farming often causes significant economic losses, which represent an obstacle in the sustainable development of culture models including IISF (Cornejo-Granados et al. 2017; Li 2020; Xiong et al. 2016; Yao et al. 2022).

Animals have evolved jointly with associated communities of microorganisms (such as in the guts), and they together are recognized as “super-organisms.” Many research reports suggested that certain microorganisms could protect aquatic animals from infection and that the gut microbiota also play an important role in the phenotypic plasticity of host (Chen et al. 2017; El-Sayed 2021). Considering the comprehensive impact of gut microbiota on the health of aquatic animal hosts, including digestion, reproductive ability, and overall immunity (Butt and Volkoff 2019), it is common in aquaculture to regulate the microbial community in aquatic organisms and use them as “beneficial partners” to overcome the challenges in aquaculture as well (Holt et al. 2021). Moreover, the gut bacteria that exhibit positive results for aquatic organisms can also serve as probiotics, preventing and protecting hosts from infection caused by pathogenic microorganisms (Yukgehnaish et al. 2020). In addition, bacterial communities are one of the main factors in an aquaculture ecosystem, which impact on nutrient cycling (Abraham et al. 2004), water quality regulation (John et al. 2020), and pathogens’ abundance (Rungrassamee et al. 2016). In aquaculture systems, environmental bacteria interact with the cultured gut bacteria species through the difference in bacterial abundance between the host and environment (Cuellar-Gempeler and Leibold 2018; Giatsis et al. 2014). Pathogenic bacteria associated with aquatic animal infections were detected in shrimp *L. vannamei* gut and rearing water, including *Vibrio*, *Pseudomonas*, and *Flavobacterium* (Hou et al. 2018). In fact, extensive studies have shown that dynamic changes in abiotic conditions, such as water temperature, pH, salinity, and inorganic nitrogen among other factors, modify the composition of rearing water microbial communities (Fan et al. 2019; Li et al. 2017; Zhang et al. 2014), which strongly change the structure of gut microbiota, and subsequently the host health and growth performance in shrimp (Dai et al. 2017; Xiong et al. 2017, 2014). In this context, the manipulation of environmental conditions might improve the shrimp intestinal microbial community, which in turn decreases the infectious bacterial abundance, avoiding antibiotic use/abuse in aquaculture systems and enhancing host resistance against pathogenic bacteria by improving intestinal barrier function (Kamada et al. 2013) and immune response (Ubeda et al. 2017).

Until now, there are numerous reports on bacterial communities’ composition in the rearing water and shrimp guts in outdoor farming models, but little is known about the bacterial communities under the industrial indoor farming models (Huang et al. 2021; Xiong et al. 2018; Zeng et al. 2017; Zhang et al. 2019). Therefore, in the present study, we used high-throughput Illumina sequencing technology (Illumina, San Diego, CA, USA) to investigate the 16S rRNA genes of the shrimp *L. vannamei* guts and rearing water bacterial communities in IISF models. We analyzed the microbial communities’ composition in shrimp guts and rearing water from six farms adopting two typical models of indoor systems. We hypothesized that bacterial composition analysis, under different environmental conditions, could reveal the relationship between shrimp guts and rearing water microbial communities in most popular indoor farming models. This could also provide a theoretical basis for biosecurity control in the industrial indoor shrimp farming.

## Materials and methods

### Sampling and experimental design

**Fig. 1:**
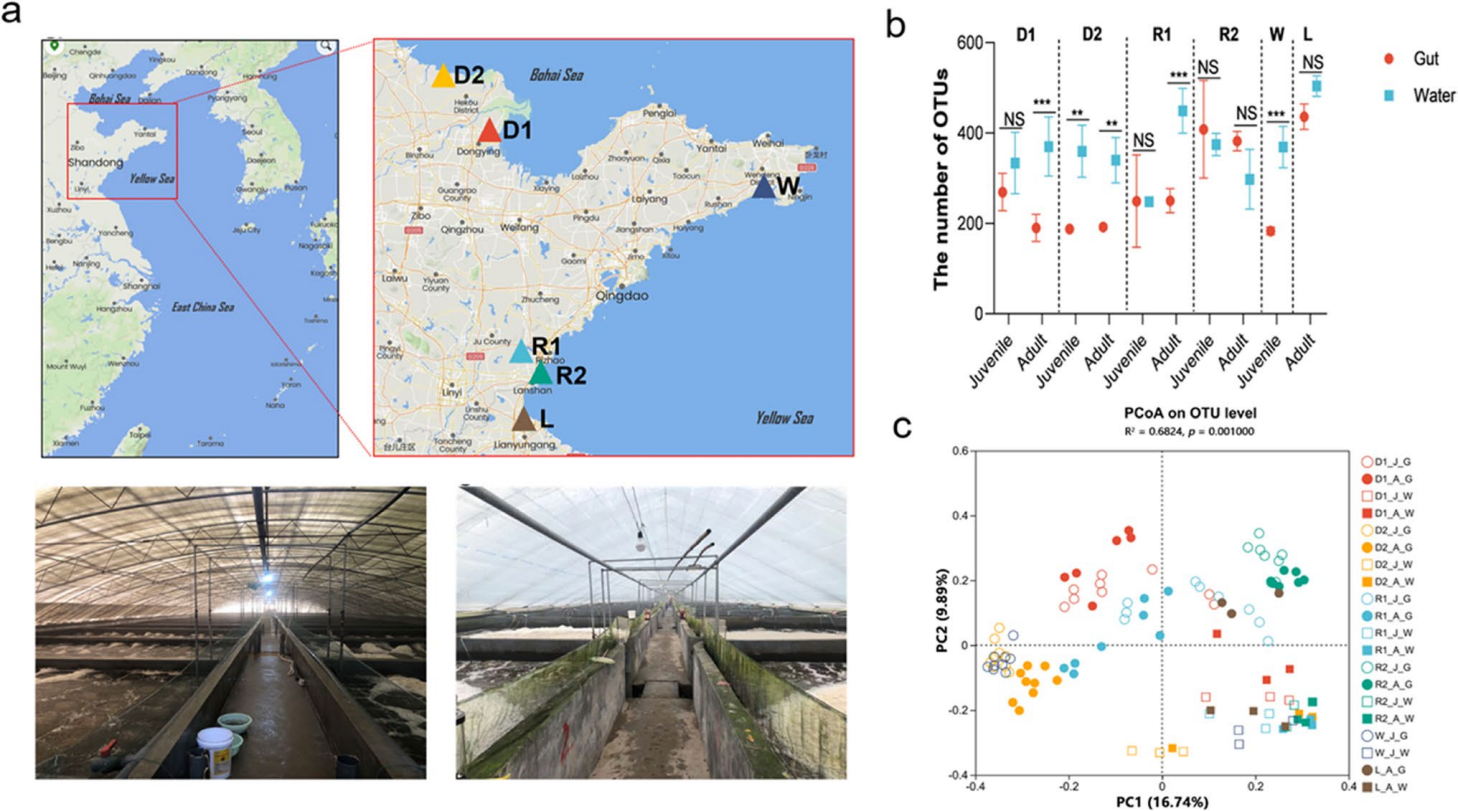
Shrimp gut microbial communities are separable by farming location and development stage. **a** Sampling locations from the selected industrial indoor farms for *L. vannamei* culture in China. **b** Number of OTUs in the shrimp *L. vannamei* gut and water samples both at the juvenile and adult stage. **c** Principal coordinate analysis (PCoA) of the shrimp *L. vannamei* gut and water microbiota composition at OTU level based on Bray–Curtis distances. The meaning of characters in labels is as follows: The first character is the abbreviation of farm location, D1 and D2 represent two farms in Dongying city of Shandong province, R1 and R2 represent two farms in Rizhao city of Shandong province, W represents a farm in Weihai city of Shandong province, and L represents a farm in Lianyungang city of Jiangsu province. The second character is the abbreviation of shrimp stage, and J represents juvenile while A represents adult. The third character is abbreviation of sample item, G and W represent gut and water, respectively. NS, not significant; *p < 0.05; **p < 0.01; ***p < 0.001

To investigate the effect of different host and environmental factors on the gut microbial composition of *L. vannamei* shrimp and rearing water, samples of shrimp and rearing water were collected from six aquafarms in northern China during January to March 2021 to represent different areas of shrimp-farming and water source. Two farms are located in Dongying city (D), Shandong province, two farms in Rizhao city (R), Shandong province, one farm in Weihai city (W), Shandong province, and one farm in Lianyungang city (L), Jiangsu province. The farming model included two types of cover materials for the production units. In D1, D2, and W farm locations, the cover material was a thick glass-wool cover decreasing the natural light penetration in the production unit. In R1, R2, and L farm locations, the cover material was a transparent plastic sheet increasing the natural light penetration to the production units (Fig. 1a). All farms followed a similar management during the culture period. The collected adult shrimps showed infection 10 days before the sampling time at the W farm location, so adult shrimps and water samples were not collected. In addition, in the D2 farm location, diseased adult shrimps included *Enterocytozoon hepatopenaei* (EHP), decapod iridescent virus (DIV1), and convert mortality nodavirus (CMNV).

The grouping for comparison analysis depended on the change in the targeted factor, and the similarity in other factors that were not targeted (Table S1 and Table S2). The comparison groups were as follows: (1) developmental stages, D1 farm location was chosen both for adult and juvenile shrimps with similar temperature, health, and light intensity; (2) health status, D1 and D2 farm locations were chosen for healthy and diseased adult shrimps with similar light and temperature conditions; (3) light intensity, R1 and D1 farm locations were chosen for low and high light intensities under similar conditions of juvenile shrimps and healthy juvenile shrimps and temperature; and (4) temperature level, R1 and R2 farm locations were chosen for temperature levels of 25 °C and 28 °C, respectively, with similar conditions of healthy adult shrimps and high light intensity.

The number of shrimp farming production tanks in D1, D2, W, R1, R2, and L are 400, 480, 80, 60, 200, and 160, respectively, and each tank area ranged from 30 to 50 m^2^. Three tanks were randomly selected from each farm location with a triple water sampling, where each tank was represented by one sampling water. At least 10 gut samples for the juveniles or 4 gut samples for the adults raised in the same tank were pooled in a bid to provide sufficient samples for sequencing analysis. For juvenile shrimp, the number of pooled gut samples was 9 (from 110 individuals), 9 (from 52 individuals), 9 (from 75 individuals), 9 (from 45 individuals), and 8 (from 69 individuals) at D1, D2, W, R1, and R2 farm locations, respectively. For adult shrimp, the number of pooled gut samples was 6 (from 26 individuals), 9 (from 36 individuals), 8 (from 36 individuals), 7 (from 22 individuals), and 3 (from 19 individuals) at D1, D2, R1, R2, and L farm locations, respectively.

After wiping the body surface of both juvenile and adult shrimp with 70% ethanol, the shrimp midgut was removed with sterile forceps and placed in a 2 ml sterile lyophilization tube, placed in liquid nitrogen, and then transferred to a refrigerator at − 80 °C for storage until analysis. A sampler with a volume of 500 mL was used to collect water samples at a depth of 50 cm in three different locations within each culture tank. One hundred milliliter of the rearing water sample was immediately filtered through by a 0.22-μm glass fiber filter membrane. Filtered membranes were transferred to a laboratory in Shanghai Majorbio Bio-Pharm Technology Co., Ltd., China, and extracted with a phenol–chloroform-isoanmyl alcohol method, succeeded by an ethanol precipitation. The remaining rearing water samples were filtered through a 0.22-μm GF/C Whatman glass fiber filter (Whatman, Maidstone, UK), and the filtrates were stored at − 20 °C until analyzation for total ammonia, nitrite, nitrate, and soluble reactive phosphate, using a continuous flow injection analyzing system (Skalar SAN^++^ System, Skalar Analytical, Breda, The Netherlands). The light intensity was measured using a D4385-01 Digital Lux Meter (Aladdin Industrial Corporation, Shanghai, China). The temperature (± 1 °C), pH, salinity (ppt), and dissolved oxygen (DO) values of rearing water were measured, using a portable YSI 556 Professional Plus multiprobe water quality meter (YSI Inc., Yellow Springs, OH, USA), and recorded at the same time for each bacterial sampling. Water quality information is found in Table S1 and S2.

### DNA extraction, amplification, and sequencing

Genomic DNA was extracted from the gut (77 samples) and rearing water samples (30 samples) using the PowerFecal® and PowerWater® DNA isolation kits (QIAGEN Sciences, Germantown, MD, USA), respectively, according to the manufacturer’s instructions. The extracted genomic DNA was checked by 1% agarose gel electrophoresis and DNA concentration, and purities were determined, with a NanoDrop 2000 UV–vis spectrophotometer (Thermo Scientific, Wilmington, NC, USA). The hypervariable region V3-V4 of the bacterial 16S rRNA genes was amplified with primer pairs 338F (5′-ACTCCTACGGGAGGCAGCAG-3′) and 806R (5′-GGACTACHVGGGTWTCTAAT-3′) by an ABI GeneAmp® 9700 PCR thermocycler (ABI, Foster City, CA, USA). The PCR product was extracted from a 2% agarose gel and purified using the AxyPrep DNA Gel Extraction Kit (Axygen Biosciences, Union City, CA, USA) according to the manufacturer’s instructions and quantified using Quantus™ Fluorometer (Promega, Madison, WI, USA). Purified amplicons were pooled in equimolar and paired-end sequenced on an Illumina MiSeq PE300 platform/NovaSeq PE250 platform (Illumina, San Diego, CA, USA), according to the standard protocols by Majorbio Bio-Pharm Technology Co. Ltd. (Shanghai, China). Then, sequencing libraries were constructed for high-throughput sequencing on the online platform of Majorbio Cloud Platform (www.majorbio.com) (Shanghai Majorbio Bio-Pharm Technology Co., Ltd.). All raw data were deposited in the NCBI sequence read archive (SRA accession number: PRJNA1012318).

### Statistical analysis

The raw 16S rRNA gene sequencing reads were demultiplexed, quality-filtered by fastp version 0.20.0 (Chen et al. 2018), and merged by FLASH version 1.2.7 (Magoč and Salzberg 2011). Operational taxonomic unit (OTU) clustering analysis and species taxonomy were performed after differentiating the samples. The software platform Usearch (vsesion 7.0.1 http: //drive5.com/uparse/) was used for OTU delineation of all sequences, and bioinformatic statistical analysis of OTUs was performed at 97% similarity level. The RDP Classifier version 2.2 (Wang et al. 2007) was used to perform taxonomic analysis of the OTU representative sequences at 97% similarity level. Alpha diversity indices of gut and water column bacterial communities were analyzed using Mothur software (version v.1.30.2 https://mothur.org/wiki/calculators/). The Welch’s *t* test was used to compare the alpha diversity indices of gut bacterial communities in shrimp, to reveal the differences among life stages and each living environment. The OTUs with 97% similarity were selected, and the Shannon indexes were calculated under different random sampling using Mothur software, and Shannon–Wiener curves were produced using R language tools (Wickham and Wickham 2007). The generation of abundance tables at each taxonomic level and the calculation of beta diversity distance were implemented by Qiime (v1.9.1 http://qiime.org/install/index.html). Principal coordinate analysis (PCoA) was constructed based on Bray–Curtis or weighted Unifrac using ordination method in R v3.3.1 to visualize the distances between groups (Team RC 2014). These analysis methods refer to the research of Ren et al. (2022), and the data visualization was done by R language tools (Wickham and Wickham 2007). Water quality and bacterial community diversity index data were analyzed by the one-way ANOVA using GraphPad Prism8.0 statistical software (GraphPad Software, Boston, MA, USA). If significances of differences (*p* < 0.05) might have been identified, Duncan’s multiple range tests were used to determine the differences between experimental treatments. SourceTracker analysis was performed to evaluate the contribution (i.e., proportion) of the rearing water to the shrimp gut bacterial community, based on a Bayesian algorithm (Knights et al. 2011). The beta nearest taxon index (βNTI) is generated by a null model test of the phylogenetic β-diversity index *β* mean nearest-taxon distance (βMNTD), and Raup-Crick metric (RCbray) is generated by a null model test of the Bray–Curtis taxonomic β-diversity index (Stegen et al. 2013).

## Results

### Diversity of bacterial communities in gut and rearing water

A total of 5,363,283 effective sequences were obtained from all samples. The number of effective bases was 2,229,913,618, and the average length was 415 bp. There were 4016 OTUs from 77 shrimp gut samples and 2137 OTUs from 30 water samples. Quantitative sequencing data were randomly selected, and the Shannon index dilution curve was constructed according to the number of OTUs that can be represented (Fig. S1). With the increase of the number of reads sampled, the curve tended to be gentle gradually, indicating that the amount of sequencing data can reflect the vast majority of microbial diversity information in the sample, which can better cover the diversity of the microbial community, and the results of microbial community analysis are reasonable.

Comparing the OTU counts of the shrimp gut and corresponding rearing water from all sampling sites, a trend was found, resulting in OTU counts of shrimp gut being slightly lower than that of rearing water. There were no significant differences in either gut and rearing water samples during the juvenile and adult shrimp stages, except for the rearing water sample during adult stage at the R1 site location (Fig. 1b).

Measures of within-sample diversity (α-diversity) revealed an overall decreased diversity gradient from the rearing water to the shrimp gut, although only the Shannon indices at adult period in D1, the Ace indices at both juveniles and adults in D2, and at adults in R1, the Chao1 indices at adult period in D1, at both juvenile and adult period in D2, at adult period in R1, and at adult period in W, were significantly different (*p* < 0.05) (Fig. S2). The Chao1 and Ace indices are commonly used to reflect the species richness within bacterial community. Relative lower indices of gut bacteria were found in the juvenile stage in D2 (Chao1 = 272.0 ± 29.7; Ace = 266.0 ± 52.4) and W (Chao1 = 260.2 ± 15.0; Ace = 292.7 ± 12.8), where serious diseases occurred in adult shrimps.

The principal coordinate analyses (PCoAs) based on the Bray–Curtis distance were performed to investigate patterns of separation between microbial communities. We have found a clear separation between bacterial communities in shrimp gut and rearing water (*p* = 0.001, *R*^2^ = 0.6824) (Fig. 1c), and the consistency of rearing water bacteria was markedly higher than that of the gut bacterial community. In the PCoAs of the total microbial community, the IISF type and farm location separate across the first principal coordinate (PC1), indicating that the largest source of variation in microbial communities of shrimp’s gut is the IISF type. It was the main factor influencing the assembly of gut bacteria, with a 16.74% explanation of variance. Moreover, the diversity of gut bacteria of shrimp raised in W was similar to that in D1 and D2, where the farming model has low light intensity, while the diversity of gut bacteria of shrimp raised in L was similar to that in R1 and R2, where the farming model has high light intensity (Fig. 1c). However, rearing water samples across different farming models were clustered in one quarter in PCoA analysis (Fig. 1c and Fig. S3).

### Shrimp gut bacterial composition

**Fig. 2:**
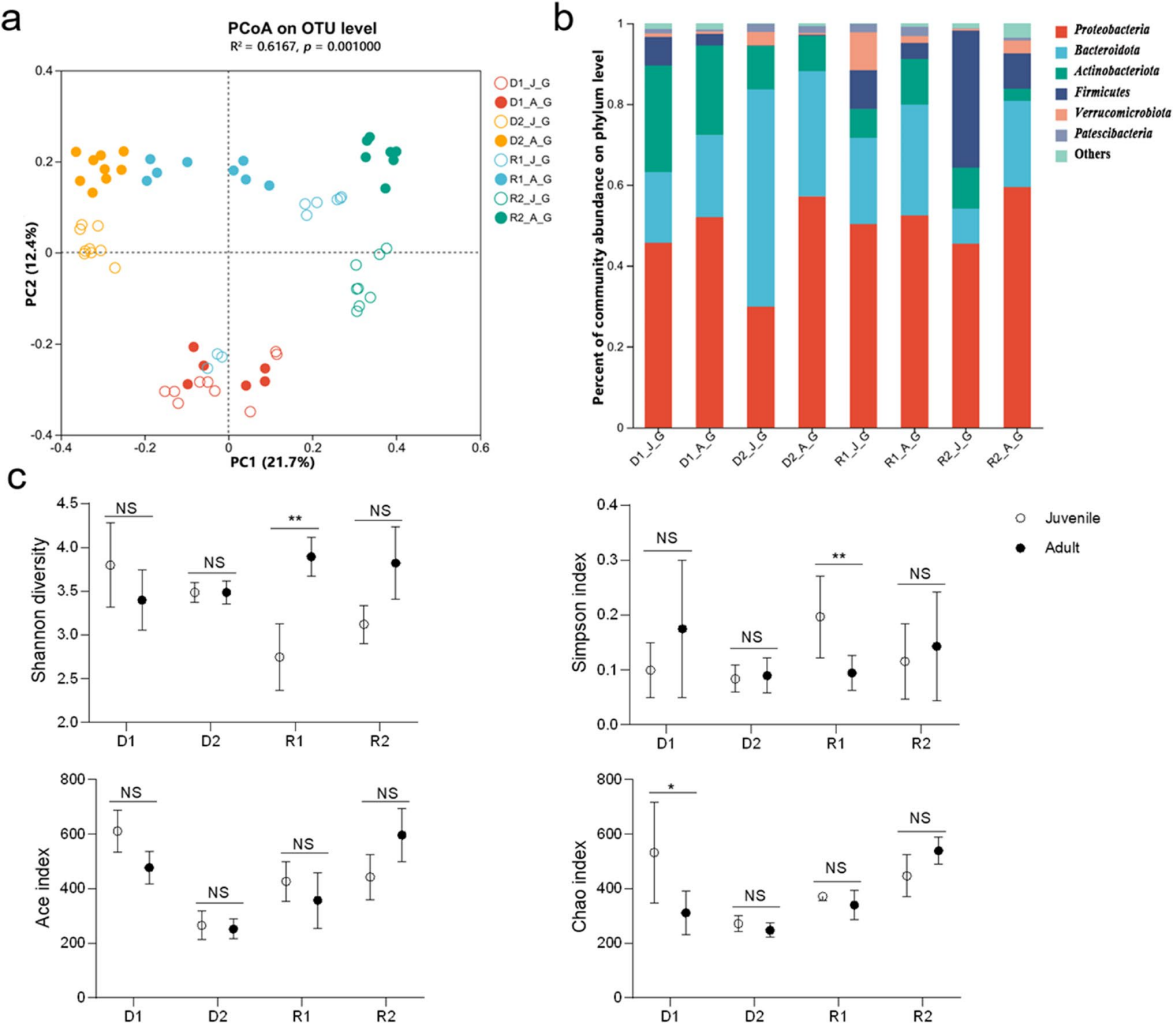
Shrimp gut microbial communites differ between the juvenile and adult stage. **a** Principal coordinate analysis (PCoA) of shrimp *L. vannamei* gut microbiota composition analysis based on Bray–Curtis distances (OTU level). **b** Bacterial community composition of juvenile and adult shrimp *L. vannamei* gut at phylum level. **c** Diversity indices of gut bacterial communities in juvenile and adult shrimp. The meaning of characters in label are as follows: The first character is the abbreviation of farm location, D1 and D2 represent two farms in Dongying city, R1 and R2 represent two farms in Rizhao city. The second character is the abbreviation of shrimp stage, and J represents juvenile while A represents adult. The third character is abbreviation of sample item, and G represents shrimp gut. NS, not significant; *p < 0.05; **p < 0.01; ***p < 0.001

In order to reveal the progression of shrimp gut bacterial communities during their grow-out period in the IISF model, we selected 65 samples of D1, D2, R1, and R2 including both juvenile and adult shrimp and performed PCoAs based on the Bray–Curtis distance. The results showed that the gut bacterial community was distributed along the PC2 axis from juvenile to adult shrimp with an explanation of 12.4%, for farms with complete culture cycles. Additionally, geographical location of farms was still the main factor contributing to the separation of gut bacterial communities with an explanation of 21.7% (Fig. 2a). Comparison of the variation in gut diversity between juvenile and adult shrimp showed that only the adult shrimp microbial diversity was significantly higher than that of juvenile shrimp in R1; in the other areas, although the adult shrimp gut microbial diversity was higher than in juvenile shrimp, the differences were not significant (Fig. 2a, c).

*Proteobacteria* was found as the first dominant phylum (45.68%), followed by *Actinobacteria* (26.36%), *Bacteroides* (17.5%), *Firmicutes* (7.10%), and *Patescibacteria* (1.12%) in the gut of juvenile shrimp in the IISF model, and corresponding adult shrimp showed a similar dominant phyla pattern with higher *Proteobacteria* and *Bacteroidota*, but lower *Actinobacteria* and *Firmicutes* (Fig. 2b, Fig. S4a, Fig. S5). At the family level, *Rhodobacteraceae* and *Flavobacteriaceae* were the dominant families both for the juveniles and adults, whereas adults showed a higher abundance in comparison with the juveniles (Fig. S4b, Fig. S5, and Fig. S6a). It is worth noting that shrimp gut at an adult stage harbored a higher abundance of *Vibrionaceae*, but a lower abundance of *Mycoplasmataceae* and *Mycobacteriaceae* than at a juvenile stage. At the genus level, a higher abundance of *unclassified_f_Rhodobacteraceae* and lower abundance of *Ruegeria* and *Mycobacterium* in adult gut were observed compared to juvenile gut, although there were a large number of unclassified genera (Fig. S4c, Fig. S6b).

### Source tracking of shrimp gut bacteria

**Fig. 3:**
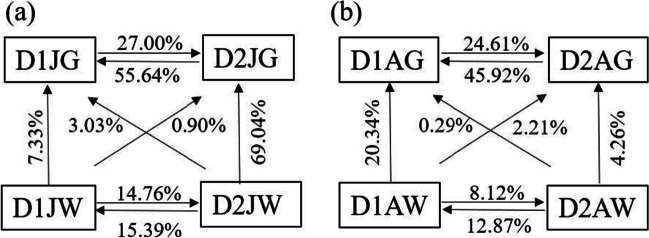
Source tracking between individual shrimp and rearing water at D1 and D2. It was estimated within a Bayesian framework in SourceTracker at shrimp juvenile (**a**) or adult (**b**) stages, respectively. D1 and D2 represent two farms in Dongying city, J and A represent juvenile and adult shrimp, respectively, G represents gut, and W represents rearing water

Source tracking analysis showed that the proportion of adult shrimp gut microbiota sourced from rearing water was 33%, 49%, 5%, and 14% in farm of D1, D2, R1, and R2, respectively (Fig. S7). The average proportion level was higher in the Dongying area than in the Rizhao area, and contribution from rearing water microbiota varied with bacterial species and shrimp age (Fig. S5 and Fig. S6). Then, the samples from Dongying area (D1 and D2), where the culture facilities for IISF models are relatively better and the rearing water condition are more stable than those in Rizhao area, were selected for further source tracking analysis. It was found that the proportion of rearing water mutual sources of bacterial communities in D1 and D2 was low (8.12 ~ 15.39%), while the shrimp gut mutual sources of bacterial communities in D1 and D2 was relatively high (24.61 ~ 55.64%). At D1, the proportion of gut bacteria originated from rearing water was increased from 7.33% at juvenile stage to 20.34% at adult stage; the proportion increased (20.34%). On the contrary, it decreased significantly from 69.04 to 4.26% at D2 (Fig. 3). This result indicated that there were different incorporation rates of bacteria in rearing water from different farming site. The relative contributions of deterministic and stochastic processes to the bacterial community assembly were further analyzed using a null model based on βNTI. The results showed that stochastic processes played a more important role than deterministic processes in the bacterial community assembly processes of rearing water. However, the relative importance of deterministic processes has been found in the gut bacterial community of both the juvenile and adult shrimp at D2, since they had a βNTI and RCbray value less than − 2 and − 0.95, respectively, among inter-group samples (Fig. S8). For the assessment of the correlation of shrimp gut bacterial community and rearing water quality, the canonical correlation analysis (CAA) was performed using a CCA program in vegan packages of R software. It was found that the total ammonia nitrogen (TAN) level in water showed the highest value for gut microbiota at the juvenile stage, and salinity and nitrate content at the adult stage (Fig. S9).

### Environmental factors impact gut bacterial diversity

To further investigate the correlation of shrimp gut bacterial community formation with various environmental factors or host health, we divided the whole sample into several comparison groups based on the matched farming conditions such as light intensity, temperature fluctuation, and disease occurrence. As to the gut bacterial diversity, shrimp raised at 25 °C showed a relatively higher OUT level. The Ace indices of gut bacterial community in shrimp raised at 28 °C, low light intensity, or infected with pathogens were significantly lower than those in shrimp raised at 25 °C, high light intensity, or without any infection, respectively (Table 1).

**Table 1:** Bacterial community diversity indices in *L. vannamei* shrimp gut

Investigated factors/factor levels		OTUs	Shannon	Simpson	Ace	Chao1
Age	Juvenile	269 ± 41a	3.27 ± 0.39a	0.07 ± 0.01a	350.28 ± 54.23a	321.47 ± 78.16a
	Adult	190 ± 30a	2.81 ± 0.61a	0.12 ± 0.04a	293.27 ± 87.21a	249.19 ± 101.51a
Health status	Healthy	190 ± 30a	2.81 ± 0.61a	0.12 ± 0.04a	293.27 ± 87.21a	249.19 ± 101.51a
	Diseased	192 ± 5a	2.80 ± 0.10a	0.10 ± 0.01a	156.99 ± 36.36b	145.55 ± 34.63a
Light intensity	High	249 ± 65a	2.75 ± 0.38a	0.20 ± 0.07a	426.90 ± 72.33a	371.30 ± 15.68a
	Low	269 ± 41a	3.27 ± 0.39a	0.07 ± 0.01a	350.28 ± 54.23b	321.47 ± 78.16a
Temperature	25 °C	436 ± 28a	3.27 ± 0.16a	0.09 ± 0.02a	362.26 ± 26.00a	363.18 ± 27.21a
	28 °C	382 ± 22b	2.55 ± 0.26a	0.16 ± 0.07a	315.13 ± 77.06b	299.36 ± 70.96b

Data with different superscripts letter within each column indicate significant differences among groups

### Light intensity impact gut bacterial composition

**Fig. 4:**
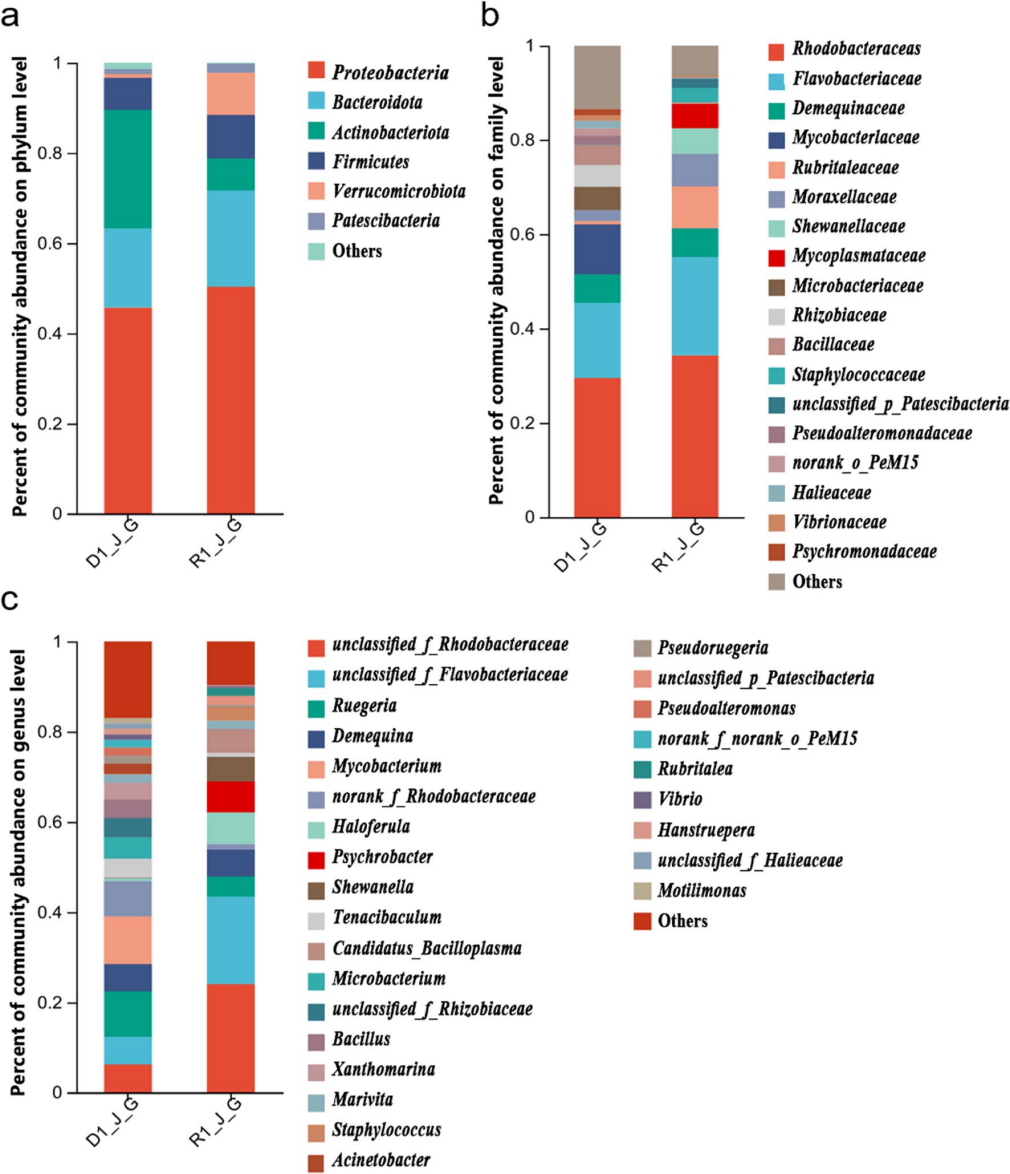
Gut bacterial community composition at phylum (**a**), family (**b**), and genus (**c**) levels for the *L. vannamei* shrimp raised under different light intensities. D1_J_G represents low light intensity A group, and R1_J_G represent high light intensity group

At the phylum level, the intestine of juvenile shrimps raised under low light intensity showed *Proteobacteria* as the dominant phylum (45.68%), in addition to *Actinobacteriota* (26.36%) and *Bacteroidota* (17.50%), exhibiting different bacterial abundances. The intestine of shrimps raised under high light intensity showed the *Proteobacteria* as the dominant phylum (50.34%), in addition to *Bacteroidota* (21.29%) and *Firmicutes* (9.61%) with different bacterial abundances (Fig. 4a and Fig. S10). It was observed that the intestine of juvenile shrimps raised under low light intensity showed a higher bacterial abundance of *Actinobacteriota* and a lower bacterial abundance of *Proteobacteria* and *Bacteroidota*, compared to those under high light intensity.

At the family level, the intestine of juvenile shrimps raised under low light intensity showed *Rhodobacteraceae* as the dominant family (29.52%), in addition to the *Flavobacteriaceae* (15.95%) and *Mycobacteriaceae* (10.58%) with different bacterial abundances. The intestine of juvenile shrimps raised under high light intensity showed *Rhodobacteraceae* as the dominant family (34.31%), in addition to *Flavobacteriaceae* (20.87%) with different bacterial abundances (Fig. 4b and Fig. S10). It was observed that the intestine of shrimps raised under low light intensity showed a higher bacterial abundance of *Pseudoalteromonadaceae* and *Vibrionaceae* and a lower bacterial abundance of *Rhodobacteraceae* and *Flavobacteriaceae,* compared to the intestine of juvenile shrimps raised under high light intensity. In fact, only *Mycobacteriaceae* and *Microbacteriaceae* were detected in the intestine of juvenile shrimp under low light intensity, while *Shewanellaceae* and *Mycoplasmataceae* were detected only in the intestine of shrimp grown under high light intensity.

At the genus level, the intestine of juvenile shrimps from tanks with low light intensity showed *Mycobacterium* as the dominant genus (10.58%), in addition to *Ruegeria* (10.07%) with different bacterial abundances. The intestine of juvenile shrimps from tanks with high light intensity showed the *unclassified_f_Rhodobacteraceae* as the dominant genus (24.01%), in addition to *unclassified_f_Flavobacteriaceae* (19.44%) with different bacterial abundances (Fig. 4c). It was observed that the intestine of juvenile shrimps raised under low light intensity showed a higher bacterial abundance of *Ruegeria* and *Vibrio*, and a lower bacterial abundance of *unclassified_f_Rhodobacteraceae* and *unclassified_f_Flavobacteriaceae* compared to those under high light intensity. *Mycobacterium*, *Bacillus*, and *Pseudoalteromonas* occur only in the intestine of juvenile shrimp raised under low light intensity, while *Shewanella* and *Psychrobacter* were detected only in the intestine of juvenile shrimp raised under high light intensity.

### Water temperature impacts gut bacterial composition

**Fig. 5:**
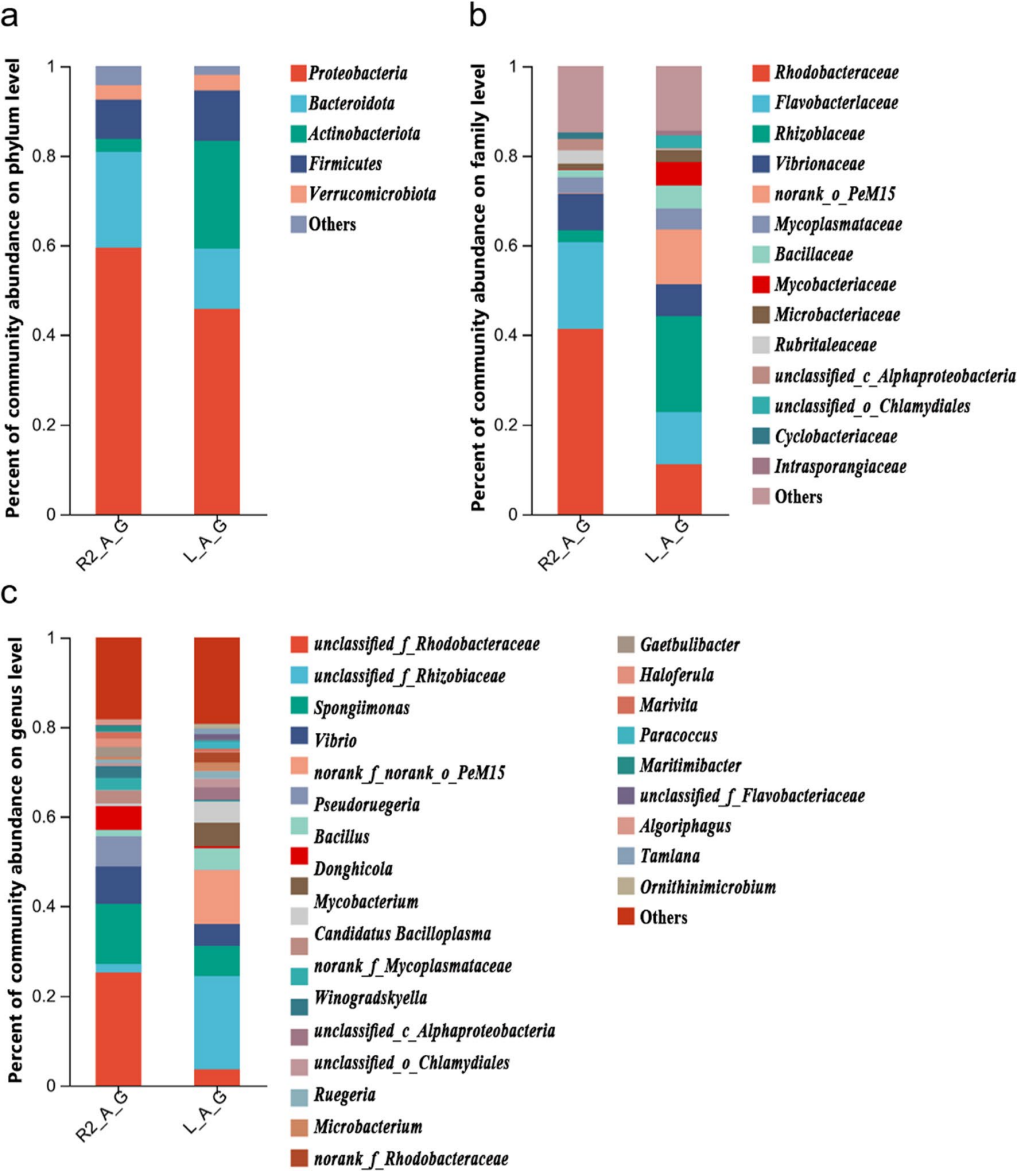
Gut bacterial community composition at phylum (**a**), family (**b**), and genus (**c**) levels for the adult shrimp *L. vannamei* raised under different temperatures (25 °C and 28 °C). R2_A_G represents 28 °C group; L_A_G represents 25 °C group

At the phylum level, the intestine of the adult shrimp group raised at a water temperature of 25 °C showed *Proteobacteria* as the dominant phylum (45.83%), in addition to *Actinobacteriota* (24.06%), with different bacterial abundances. The intestine of adult shrimps raised at 28 °C showed *Proteobacteria* as the dominant phylum (59.51%), in addition to *Bacteroidota* (21.31%), with different bacterial abundances (Fig. 5a). It was observed that the intestine of shrimps raised at 28 °C showed a higher bacterial abundance of *Proteobacteria* and *Bacteroidota* and a lower bacterial abundance of *Actinobacteriota* and *Firmicutes,* compared to the intestine of adult shrimp raised at 25 °C.

At the family level, the intestine of adult shrimp raised at 25 °C showed *Rhizobiaceae* as the dominant family (21.44%), in addition to *the norank_o_PeM15* (12.11%), with different bacterial abundances. The intestine of adult shrimp raised at 28 °C showed *Rhodobacteraceae* as the dominant family (41.41%), in addition to *Flavobacteriaceae* (19.21%) and *Vibrionaceae* (8.31%), with different bacterial abundances (Fig. 5b). It is observed that the intestine of adult shrimp raised at 28 °C showed a higher bacterial abundance of *Rhodobacteraceae*, *Flavobacteriaceae,* and *Vibrionaceae*, and a lower bacterial abundance of *Rhizobiaceae* and *Microbacteriaceae*, compared to the intestine of adult shrimp raised at 25 °C. In fact, *Mycobacteriaceae* was only detected in the shrimp of the 25 °C group.

At the genus level, the intestine of adult shrimp raised at 25 °C showed the *unclassified_f__Rhizobiaceae* as a dominant genus (20.89%). The intestine of the 28 °C group showed the *unclassified_f__Rhodobacteraceae* as the dominant genus (25.18%), in addition to *Spongiimonas* (13.44%), with different bacterial abundances (Fig. 5c). It was observed that the intestine of adult shrimp raised at 28 °C showed a higher bacterial abundance of *unclassified_f__Rhodobacteraceae* and *Vibrio seudoruegeria*, and a lower bacterial abundance of *unclassified_f__Flavobacteriaceae* and *norank_f__Mycoplasmataceae*, compared to the adult shrimp of the 25 °C group. In fact, *Mycobacterium* and *Photobacterium* were only detected in the shrimp of the 25 °C group, and *Pseudoruegeria* was only detected at the adult shrimp group raised at 28 °C.

### Shrimp health status impacts gut bacterial composition

**Fig. 6:**
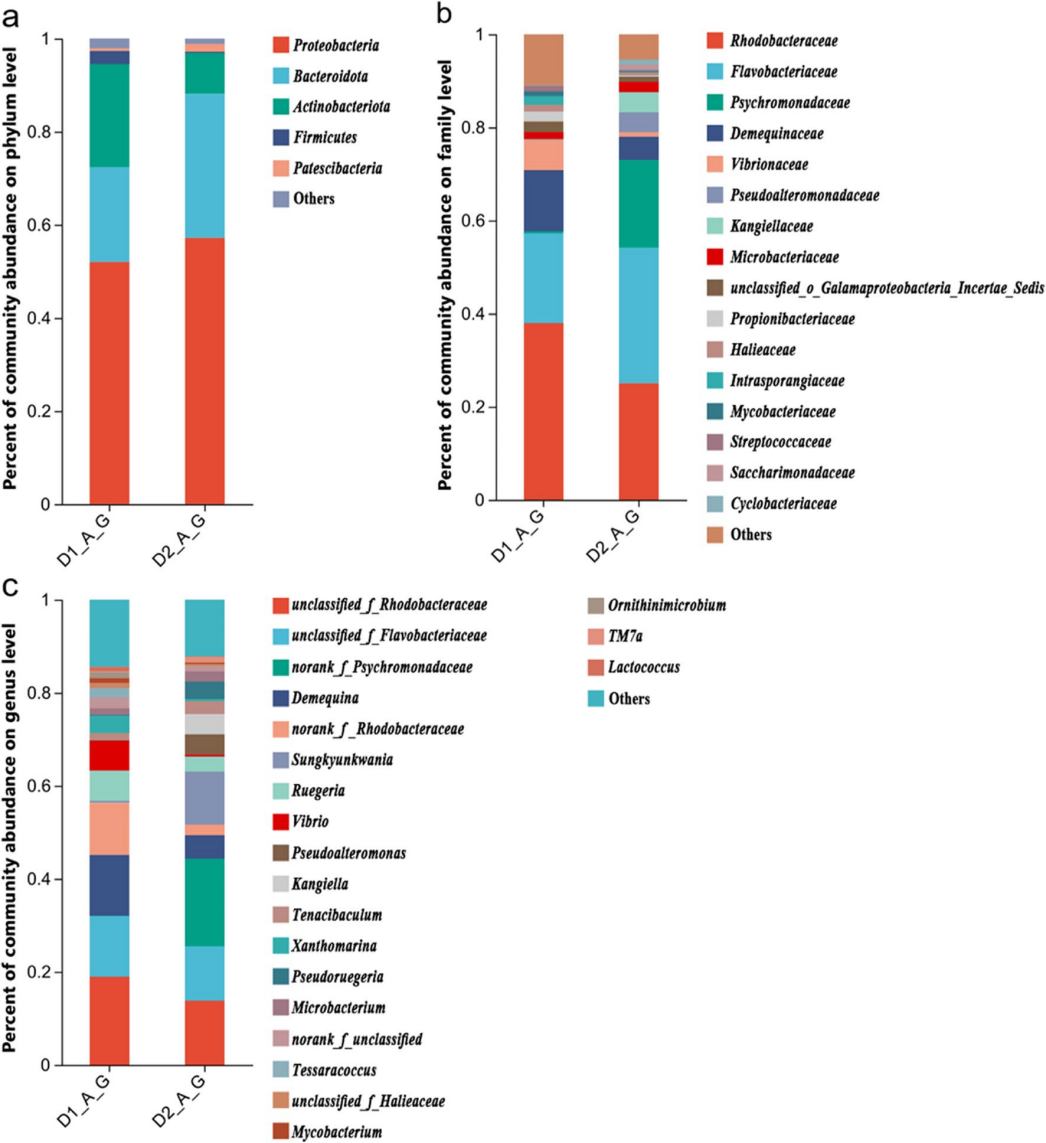
Gut bacterial community composition at phylum (**a**), family (**b**), and genus (**c**) levels for the healthy and infected adult shrimp *L. vannamei*. D1_A_G represents healthy shrimp; D2_A_G represents diseased shrimp

At the phylum level, the intestine of healthy adult shrimp showed *Proteobacteria* as the dominant phylum (52.02%), in addition to *Actinobacteria* (22.14%) and *Bacteroides* (20.35%), with different bacterial abundances. The intestine of adult shrimp with infection showed *Proteobacteria* as the dominant phylum (57.19%), in addition to *Bacteroides* (30.93%), with different bacterial abundances (Fig. 6a).

At the family level, the intestine of healthy adult shrimp showed *Rhodobacteraceae* as the dominant family (37.91%), in addition to *Flavobacteriaceae* (19.33%) and *Demequinaceae* (13.09%), with different bacterial abundances. The intestine of adult shrimp with infection showed *Flavobacteriaceae* as the dominant family (29.09%), in addition to *Rhodobacteraceae* (25.03%) and *Psychromonadaceae* (18.85%) (Fig. 6b). At the genus level, the intestine of healthy adult shrimp showed the *unclassified_f__Rhodobacteraceae* as the dominant genus (18.89%), in addition to *Demequina* (13.09%), *unclassified_f__Flavobacteriacea* (12.97%), and *norank_f__Rhodobacteraceae* (11.16%), with different bacterial abundances. The intestine of adult shrimp with infection showed *norank_f_Psychromonadaceae* as the dominant genus (18.85%), in addition to *unclassified_f_Rhodobacteraceae* (13.78%), *unclassified_f_Flavobacteriacea* (11.69%), and *Sungkyunkwania* (11.37%), with different bacterial abundances (Fig. 6c).

### Specific functional bacteria in shrimp gut

Shifts in intestinal microbiota could indicate the health status of a host. The intestine of juvenile shrimp showed a higher *Pseudoalteromonadaceae* abundance and a higher *Firmicutes*/*Bacteroidota* ratio (F/B), compared to adult shrimp, while the intestine of adult shrimp showed a higher abundance of *Vibrionaceae*. Healthy adult shrimp showed a higher *Vibrionaceae* abundance and a higher F/B ratio, compared to infected adult shrimp, while the intestine of infected adult shrimp showed a higher abundance of *Pseudoalteromonadaceae* compared to the healthy ones. In adult shrimp cultured at 28 °C, a higher *Vibrionaceae* abundance and a lower F/B ratio were observed compared to the intestine of adult shrimp with water temperature of 25 °C (Table 2).

**Table 2:** The abundance of four functional bacteria in the *L. vannamei* shrimp gut

Investigated factor level	Habitat	Bacterial abundance (%)				
		*Bacteroidota* (B)	*Firmicutes *(F)	*Vibrionaceae*	*Pseudoalteromonadaceae*	F/B ratio
Juvenile low light 28 °C	Gut	17.5	7.1	0	1.77	40.5
Juvenile high light 28 °C	Gut	21.29	9.61	0.38	0	2.2
Healthy adult High light 28 °C	Gut	20.35	2.84	6.54	0	13.9
Diseased adult high light 28 °C	Gut	30.93	0.23	0.95	4.30	0.74
25 °C adult high light	Gut	13.41	11.34	7.17	0	84.5
28 °C adult high light	Gut	21.31	8.78	8.31	0	41.2

In addition, the abundance of *Pseudoalteromonadaceae* and F/B ratio in the intestine of the juvenile shrimp group grown under low light intensity were higher than those of juvenile shrimps grown under high light intensity (Table 2).

## Discussion

Gut bacterial community is called an “extra organ” in the host regarding their beneficial effects on digestive efficiency, organism’s health, and organism’s immunity (Ai et al. 2018; Ge et al. 2018). Considering the importance of gut microbiota in animal health and nutrition, manipulation of beneficial microbial communities in shrimp gut may provide a solution for the improvement of production performance and resistance to pathogens (Landsman et al. 2019a). However, the processes of formation and the progression of shrimp gut microbiota communities are really sophisticated processes, and no dominant driving factors have been found along shrimp growth period, since both the host genetics and environmental conditions, such as water temperature, salinity, sulfide concentration, practice mode, and disease as well have shown their effects (Holt et al. 2021; Landsman et al. 2019a; 2019b; Xiong et al. 2014; 2018; 2019). In fact, previous reports have shown that shrimp juveniles are more sensitive to the bacterial change compared to the adult individuals (Xiong et al. 2018; Yan et al. 2016). In our study, health status, light condition, and temperature levels were more positively effective factors on the host microbial composition compared to the age factor.

Previous studies revealed that bacterial communities exhibit a significant difference between shrimp gut and rearing water (Cornejo-Granados et al. 2017; Hou et al. 2018; Zhao et al. 2018). In our study, PCoA analysis showed that all water samples from different environmental conditions were nearly clustered and distanced from the distributed shrimp gut samples, implying a high dissimilarity between water and gut microbiota composition. This could be explained by the low microbial selective pressure between shrimp gut and rearing water environments (Tepaamorndech et al. 2020), in addition to the host modulates its own gut bacterial composition. Conversely, host gut and rearing water samples showed a similar distribution in the PCoA analysis in the outdoor pond system (Xiong et al. 2019), which could be attributed to the effect of natural environmental conditions, since these conditions were differed along production periods. In our study, the indoor farming conditions excluded the effect of the changeable environmental factors under outdoor farming conditions; this highlights the effect of developmental stage and health status on the gut microbial composition under controlled environmental conditions.

*Proteobacteria* are highly diverse and have been widely presented in aquatic invertebrate guts, usually being dominant components of bacterial community in crustaceans (Holt et al. 2021). It has been reported that *Alphaproteobacteria* dominated the stomach of healthy *L. vannamei* shrimp, while *Gammaproteobacteria* dominated the gut of both *L. vannamei* and *Penaeus monodon* shrimp (Holt et al. 2021). Our results have also shown that *Alphaproteobacteria* is the dominant class and the relative percentage of the *Gammaproteobacteria* class is higher than 10%. Furthermore, the shrimp developmental stage changes the gut microbiota composition (Cornejo-Granados et al. 2018; Zeng et al. 2017), while different growth stages show a core microbiota composition in zebrafish (Roeselers et al. 2011). A few bacterial families including *Rhodobacteraceae*, *Flavobacteriaceae*, and *Demequinaceae* showed a change in bacterial abundances along with the age change (Zhang et al. 2021). Consistent with the abovementioned, in our results, *Rhodobacteraceae* and *Flavobacteriaceae* have also been found to be dominant in juvenile and adult shrimp individuals, while the abundance of these two bacteria was higher in adult shrimp compared to juvenile individuals. *Rhodobacteraceae* and *Flavobacteriaceae* may be potential core gut microbes in the gut of the *L. vannamei* shrimp.

Gut microbiota plays a key role in host health status by maintaining the function of the intestinal barrier as it is a main gate to the internal tissues (Cabello 2006; Clemente et al. 2012; Frank et al. 2007). Bacterial diversity plays an important role in a bacterial community function, since low diversity may lead to an increased opportunity of disease occurrence (Jones and Lennon 2010).

The intestinal bacteria are in constant competition on nutrient and space. The abundance level of each species is shaped by their active metabolism and active antagonism. After stress exposure (for example, inflammation or antibiotic treatment), the pathogens adapted quickly with possible negative effect on host health status (Sorbara and Pamer 2019). The healthy microbiota uses some strategies to overcome the pathogenic abundance. They metabolize the bile salts into secondary bile salts which they have negative effect on the pathogenic bacterial growth. The microbiota ferments dietary fibers producing short-chain fatty acids which modulate the intestinal pH and subsequently the physical structure of intestinal wall as it is the main gate for pathogens to the internal organs (Sorbara and Pamer 2019). On the other side, the pathogens target the microbiota through their secreted bacteriocin proteins. At higher level of pathogenic abundance, the secreted amount of bacterial virulence factors can drive a host inflammatory response. This response releases nitrates and oxygen molecules into the gut lumen forming an oxidative environment. In this environment, novel metabolites are formed including tetrathionate. Those released (O_2_ and NO_3_) and new formed molecules are used in pathogens’ respiration pathways as electron acceptors (Sorbara and Pamer 2019).

Xiong et al. (2015) used Illumina sequencing to compare the diversity of the gut of healthy and diseased in *L. vannamei* shrimp, and found that the bacterial phylogenetic diversity and α-diversity of the gut of diseased shrimp were lower than those of healthy shrimp. The results of the present study are consistent with those results, indicating that bacterial diversity has an important regulatory role in the intestine of cultured shrimp. In our study, the Chao1 and Ace index values were relatively low in diseased adult shrimp guts in several farm locations, which is consistent with the above studies and illustrates the significant regulatory role of bacterial diversity in the gut of cultured animals.

Healthy shrimps show a dominance of the *Rhodobacteraceae* family, whereas diseased shrimps show a dominance of *Pseudoalteromonadaceae* and *Flavobacteriaceae* families (Quinn et al. 2013; Thitamadee et al. 2016; Xiong et al. 2015). In our study, shrimp showed the same dominant bacterial families regarding shrimp health status. *Rhodobacteraceae* might be considered as probiotic bacteria in shrimp (Li et al. 2018; Liu et al. 2019), since their antimicrobial substances allow to resist the infectious bacteria (D'Alvise et al. 2014), as well as promoting the effect of produced vitamins and other nutrients (Sonnenschein et al. 2017). In the case of the *Pseudoalteromonadaceae* family, it has been known as an opportunistic pathogen in shrimp (Thitamadee et al. 2016; Xiong et al. 2015).

Light condition can exert an influence on the shrimp physiological status directly by affecting feeding and growth rates, and indirectly by affecting oxygen consumption and oxygen production by planktonic bacteria in the water column (Baloi et al. 2013; Fleckenstein et al. 2019; Khoa et al. 2020). Microalgae produce inhibitory compounds affecting the pathogens’ activities (Molina-Cárdenas and Sánchez-Saavedra 2017). Algae also disturb bacterial quorum sensing communication (acyl-homoserine lactones) weakening their ability to form bacterial biofilm, and subsequently less colonizing ability of acyl-homoserine lactones regulates the virulence of many pathogenic bacteria (Natrah et al. 2011). In parallel, the bacteria induce microalgal growth by producing vitamins, idole-3acetic-acid, and other inorganic nutrients (Molina-Cárdenas and Sánchez-Saavedra 2017).

In our study, the gut of shrimps from tanks with high light intensity had an increased abundance of *Vibrionaceae*. This could be explained by the effect of different diets and/or different shrimp genetic makeups among tanks (Landsman et al. 2019a,b), since host phylogeny could determine the bacterial composition in the gut (Roeselers et al. 2011; Sullam et al. 2012). However, the salinity of the water column is not exactly the same under high and low light intensity conditions. Thus, it is also possible that differences in salinity might have an additional effect on the bacterial composition, since in the black tiger shrimp (*Penaeus monodon*) gut, the rearing water microbial composition changed under different salinity levels (Chaiyapechara et al. 2022). Accordingly, future studies are required to clarify the effect of light intensity and salinity interaction on the microbial composition in shrimps’ gut and rearing water.

Temperature affects intestine sensitivity to certain pathogenic bacteria, e.g., high temperature increases *Vibrionaceae* abundance in *L. vannamei* intestine (Al-Masqari et al. 2022). Consistently, in the present study, *Vibrionaceae* abundance was higher at 28 °C compared to 25 °C. In terms of “functional bacteria,” as they are recognized by their effect in aquaculture literature, *Vibrionaceae* are associated with different biological functions (Yu et al. 2018), whereas *Firmicutes* are negatively associated with the number of pathogenic bacteria (Mulder et al. 2009). In fact, the *Firmicutes* (F) and *Bacteroides* (B) are involved in fermentation activity (Gillilland et al. 2012), and these bacteria modify fatty acid uptake in zebrafish (Semova et al. 2012). In humans, *Firmicutes* are associated with energy metabolism (Fan and Li 2019), and F/B is associated with disease sensitivity (Mariat et al. 2009). The results of our present study show that gut samples of adult shrimp, healthy adult shrimp, adult shrimp raised at 28 °C, and juvenile shrimp raised under high light intensity showed a higher abundance of *Vibrionaceae* and a lower abundance of *Pseudoaltromonadaceae* bacteria, compared to juvenile shrimp, diseased adult shrimp, adult shrimp raised at 25 °C, and juvenile shrimp raised under low light intensity. Additionally, gut samples of juvenile shrimp, healthy adult shrimp, adult shrimp at 25 °C, and juvenile shrimp under low light intensity showed a higher F/B ratio compared to adult shrimp, diseased adult shrimp, adult shrimp at 28 °C, and juvenile shrimp under high light intensity. For the comparison of *Firmicutes* bacteria, the abundance of this bacterium was higher in the gut samples under independent treatments in juvenile shrimp, high light intensity, and 28 °C groups. Previous findings suggest that an increase in *Firmicutes* bacteria is beneficial to the host, as it implies that there are fewer pathogenic bacteria in the organism; however, an increase in F/B values may be associated with obesity (Grigor'eva 2021). Based on the above, it can be assumed that juvenile shrimps are more sensitive to bacterial infection, and that a temperature of 28 °C and high light intensity environmental conditions may be more beneficial worthy of reference in the aquatic farming production.

The bacterial diversity was lower in rearing water, compared to the gut microbial community of *L. vannamei* in the industrial indoor farming systems. The gut microbial composition was influenced by the developmental stage, host health status, light intensity, and rearing water temperature. Juveniles were more sensitive to the infectious bacteria, while rearing water temperature of 28 °C and high light intensity conditions were major factors improving the shrimp intestinal bacterial composition. Indoor systems can be improved by regulating light level, temperature, and a continuous monitoring of the microbiota both in rearing water and in gut. Since many sequences are obtained from samples, maybe a good possibility is to focus on the sequencing analysis in *Rhodobacteraceae* family (as probiotic bacteria), *Pseudoalteromonasceae* family (as an opportunistic pathogen in shrimp), and *Firmicutes* (associated with pathogenic bacteria) as a simple tool to evaluate “farm health.” These findings suggest a decrease in the use of chemicals and antibiotics against infections in shrimp aquaculture systems for cleaner production. Future studies are required to consider the effects of shrimp genetics, dietary composition, and shrimp physiological status on the gut microbial composition.

## Supplementary Information

Below is the link to the electronic supplementary material.

Supplementary file1 (PDF 1868 KB)

## Data Availability

Data will be made available on request.
