# Supplementary material for: Profile of the gut microbiota of Pacific white shrimp under industrial indoor farming system
Source: Appl Microbiol Biotechnol. 2024 Feb 20;108:225. doi: 10.1007/s00253-024-13046-0 (PMC10879296; doi:10.1007/s00253-024-13046-0)
Supplement: Supplementary file 1 [file 253_2024_13046_MOESM1_ESM.pdf]

Supplementary Information

Analysis of the gut microbiota of Pacific white shrimp under industrial indoor farming system

Meng Li[a,b,#], Abdallah Ghonimy[b,c,#], Dai-Qiang Chen[a,b], Jitao Li[b,c], Yuying He[b,c], Laura Susana López Greco[d], Fernando Dyzenchauz[d], Zhi-Qiang Chang[b,c,*]

[a] College of Fisheries and Life Science, Shanghai Ocean University, Shanghai, 201306, PR China.

[b] Key Laboratory of Sustainable Development of Marine Fisheries, Key Laboratory of Maricultural Organism Disease Control, Ministry of Agriculture and Rural Affairs, Yellow Sea Fisheries Research Institute, Chinese Academy of Fishery Sciences, Qingdao, 266071, PR China.

[c] Function Laboratory for Marine Fisheries Science and Food Production Processes, Laoshan Laboratory, Qingdao, 266071, PR China.

[d] Universidad de Buenos Aires, CONICET, Instituto de Biodiversidad y Biología Experimental y Aplicada (IBBEA), Facultad de Ciencias Exactas y Naturales, Departamento de Biodiversidad y Biología Experimental, Laboratorio de Biología de la Reproducción y el Crecimiento de Crustáceos Decápodos, Buenos Aires, 1428EGA, Argentina.

*Corresponding author: Zhi-Qiang Chang, E-mail address: changzq@ysfri.ac.cn, Tel: +86-532-85826690.

[#]These two authors contributed equally: Meng Li and Abdallah Ghonimy.

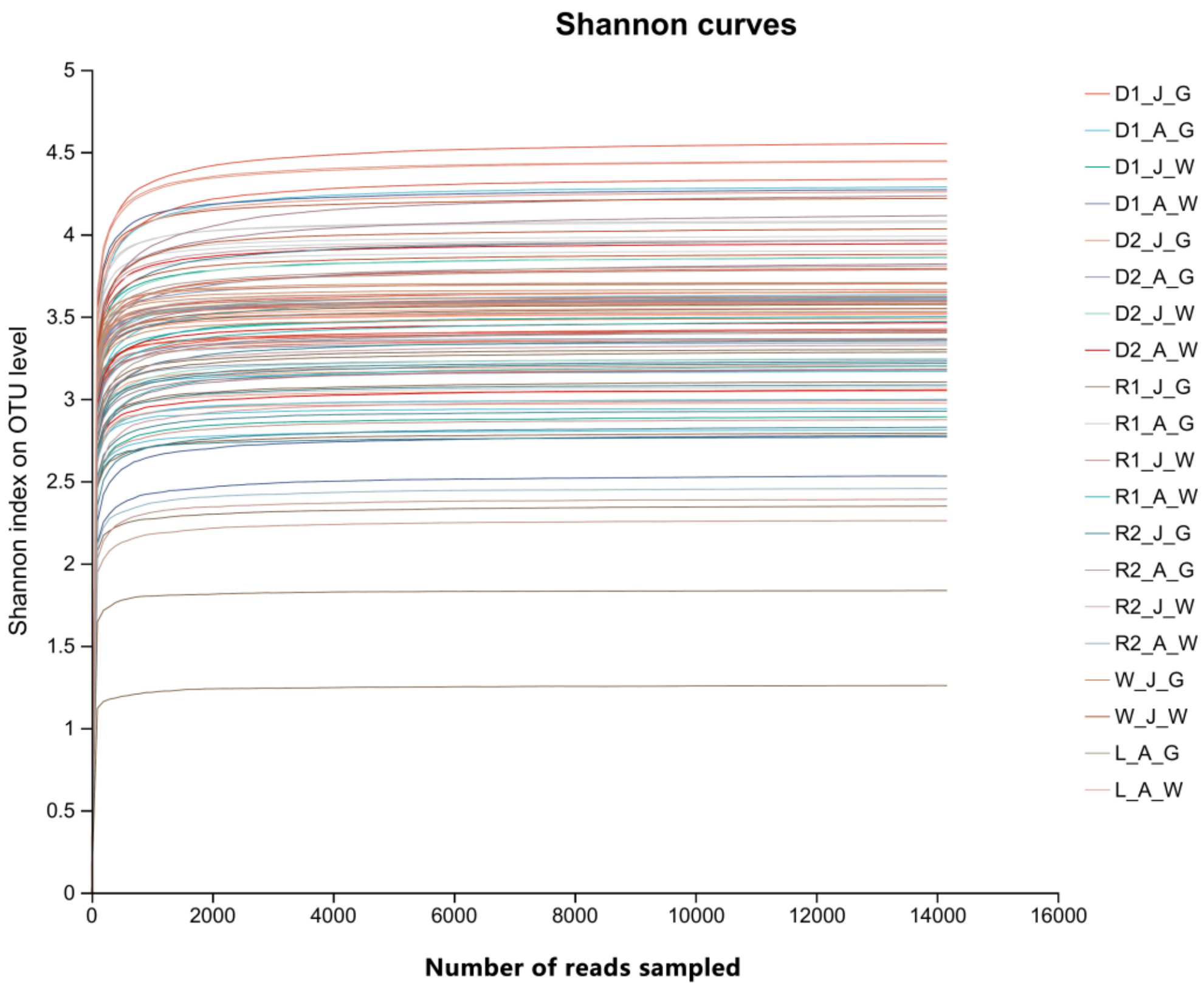

Fig. S1 Shannon's index dilution curve of samples of *L. vannamei* shrimp gut or corresponding rearing water. D1 and D2 represent two farms in Dongying city; R1 and R2 represent two farms in Rizhao city; W represents Weihai city; and L represents Lianyungang city. J and A represent juvenile and adult, respectively. G and W represent the gut and water, respectively.

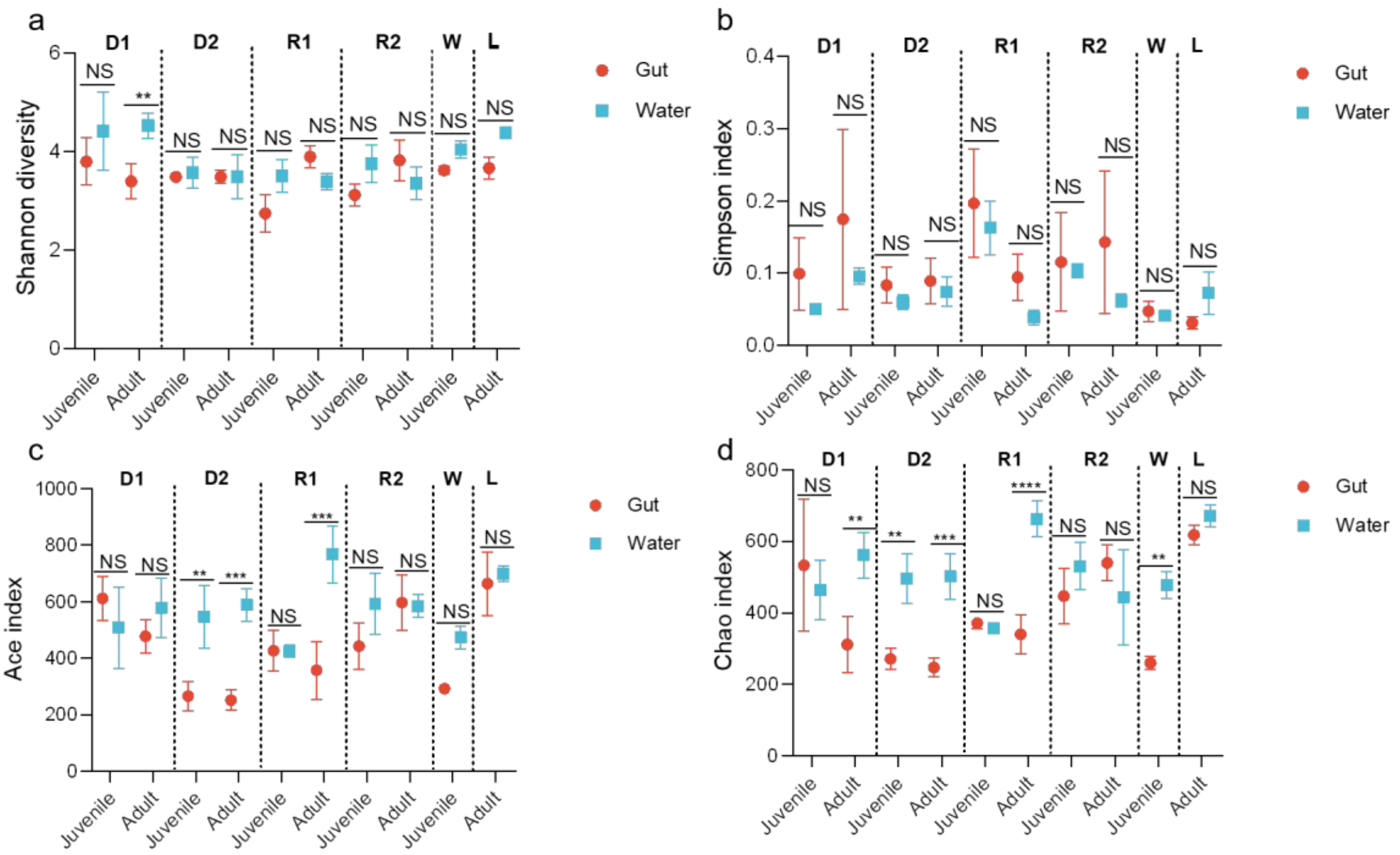

Fig. S2 Bacterial community diversity indices in the gut and water of *L. vannamei* juveniles and adults at culture periods. D1 and D2 represent two farms in Dongying city; R1 and R2 represens two farms in Rizhao city; W represents Weihai city; L represents Lianyungang city; J and A represent juvenile and adult shrimp, respectively. Circles and squares represent shrimp gut and culture water, respectively. NS: not significant; *: $p < 0.05$; * *: $p < 0.01$; * * *: $p < 0.001$.

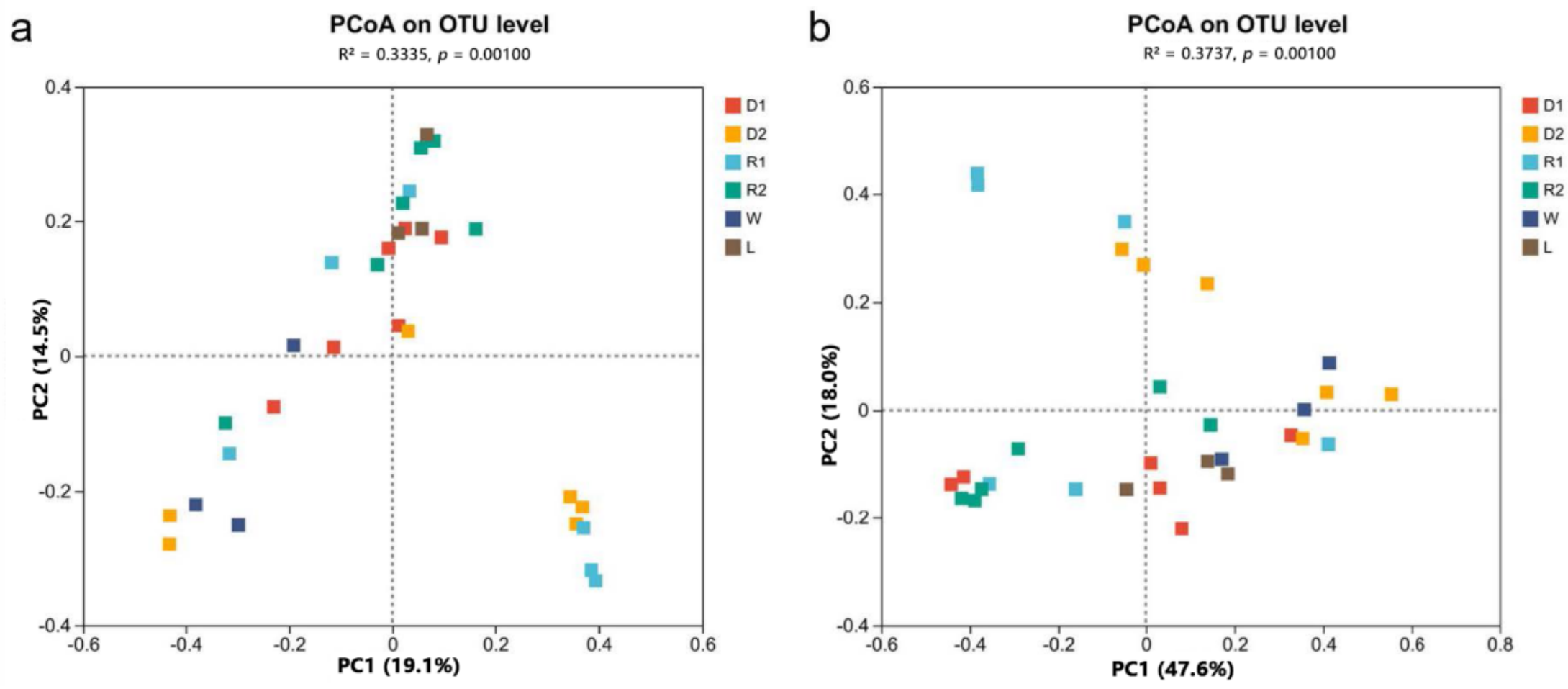

Fig. S3 Principal coordinate analysis (PCoA) of the rearing water microbiota at each farm location based on Bray-Curtis (a) or Weighted-Unifrac (b) distances at OTU level. D1 and D2 represent two farms in Dongying city; R1 and R2 represent two farms in Rizhao city; W represents Weihai city; L represents Lianyungang city.

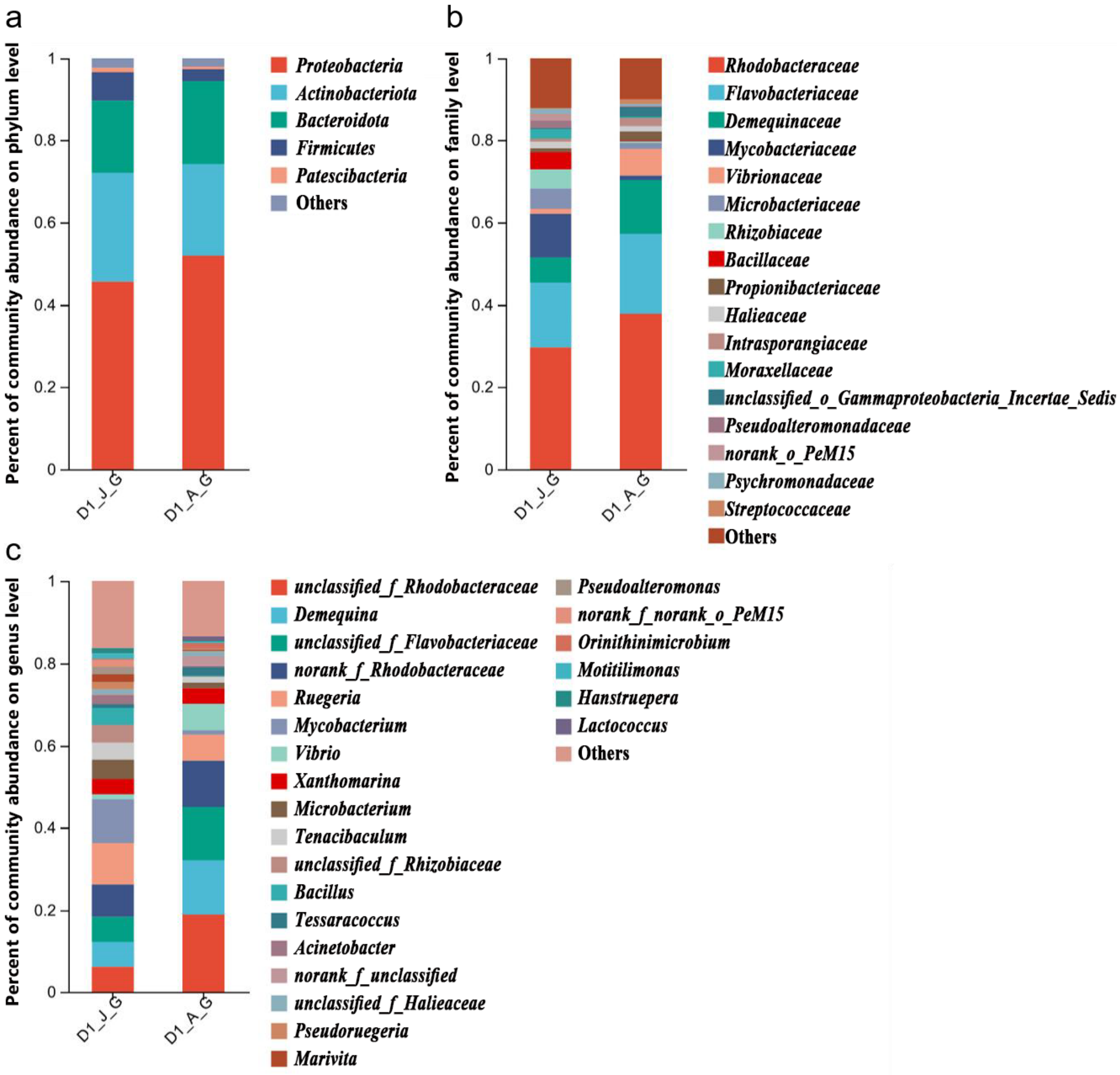

Fig. S4 Bacterial community composition of juvenile and adult shrimp *L. vannamei* gut at phylum (a), family (b), or genus levels (c), respectively. The meaning of characters in label are as follows: The first character is the abbreviation of farm location, and D represents Dongying. The second character is the abbreviation of shrimp stage, and J represents juvenile while A represents adult. The third character is abbreviation of sample item, and G represents shrimp gut.

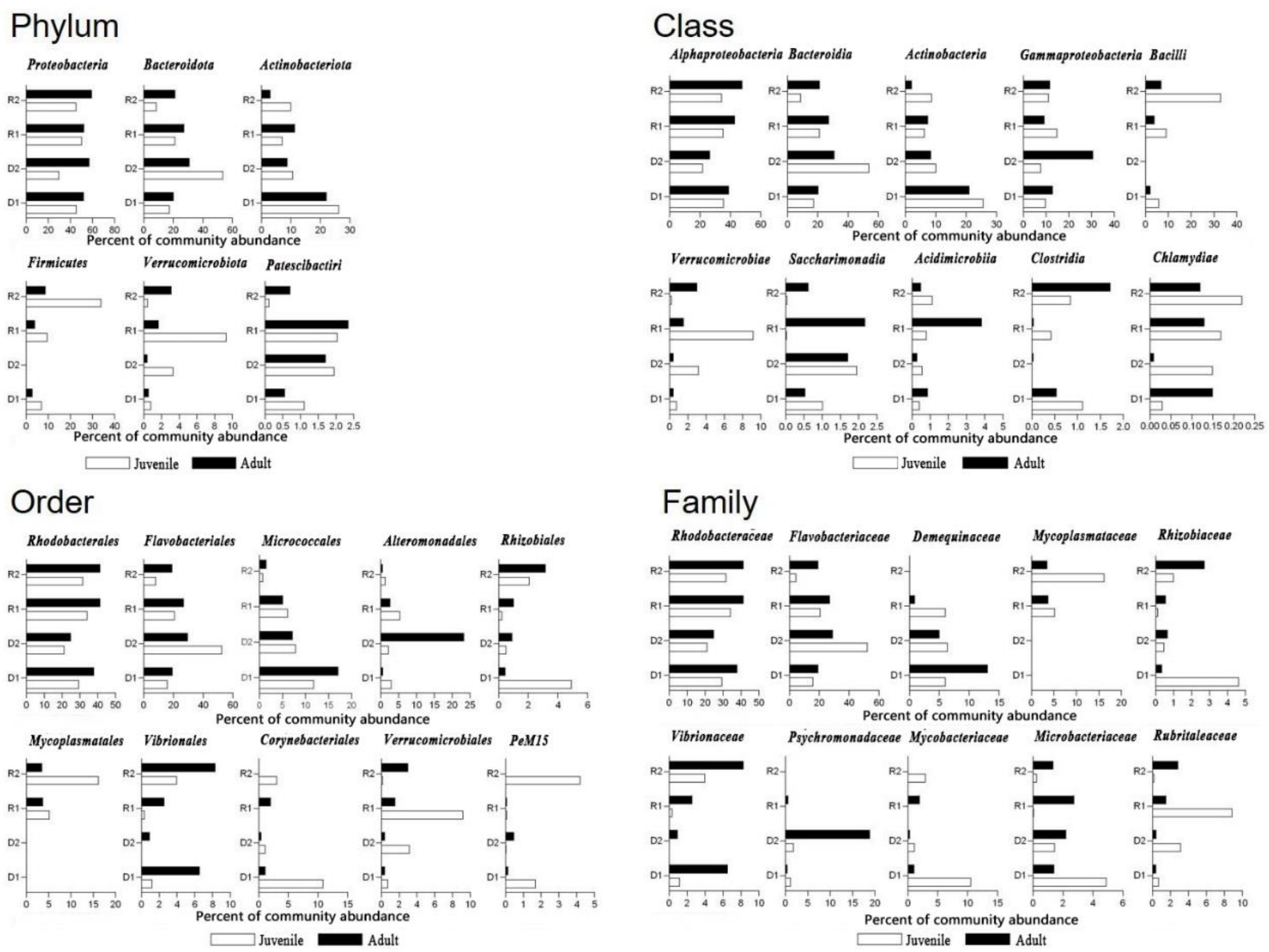

Fig. S5 Comparison of core gut bacterial community of the juvenile and adult *L. vannamei*, raised in D1, D2, R1, and R2, at phylum, class, order and family levels, respectively. D1 and D2 represent two farms in Dongying city; R1 and R2 represent two farms in Rizhao city.

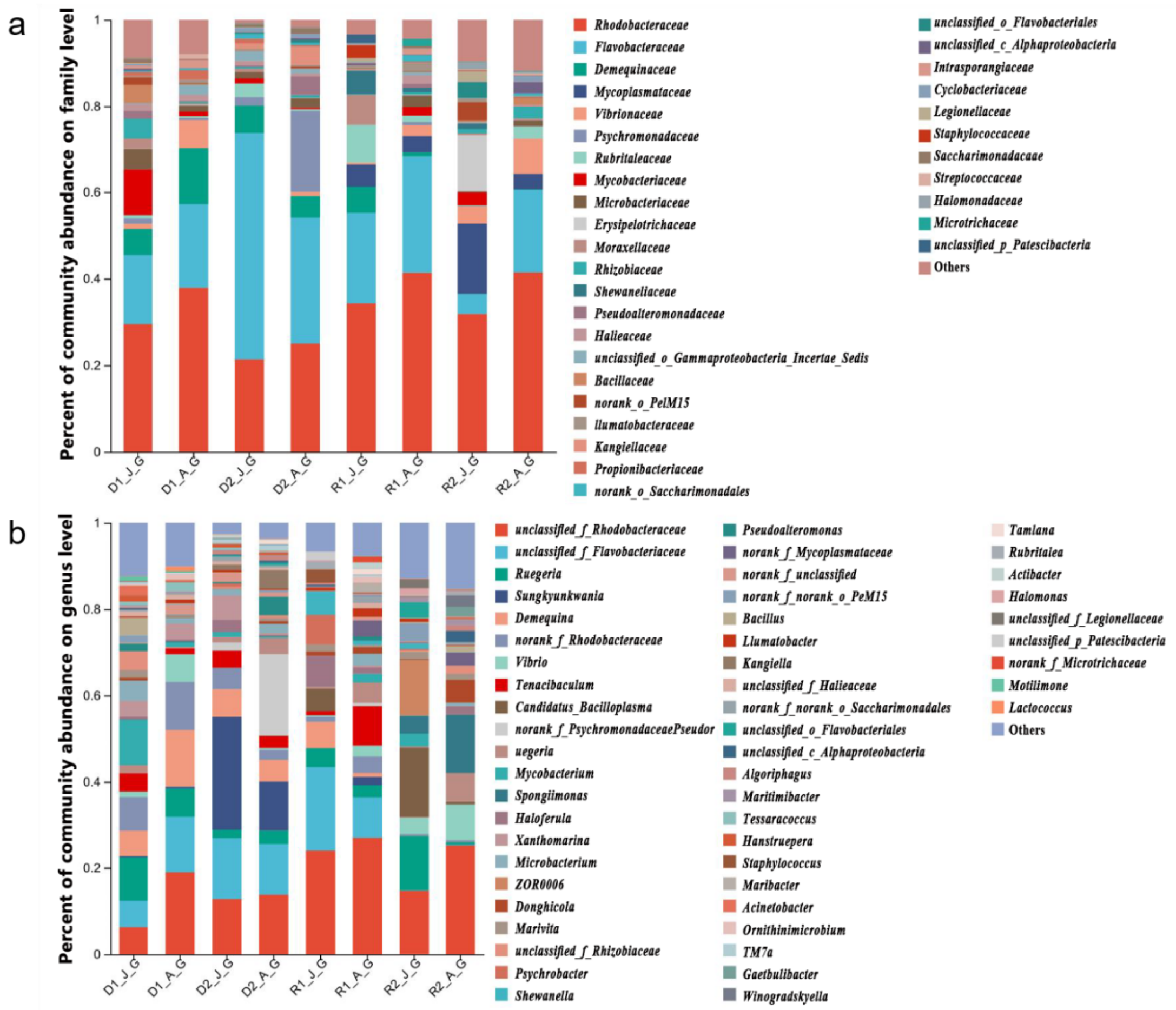

Fig. S6 Total composition on gut bacterial community of juvenile and adult *L. vannamei*, cultured in D1, D2, R1, and R2, at family (a), or genus levels (b), respectively. The meaning of characters in label are as follows: The first character is the abbreviation of farm location, D1 and D2 represent two farms in Dongying city; R1 and R2 represent two farms in Rizhao city. The second character is the abbreviation of shrimp stage, and J represents juvenile while A represents adult. The third character is abbreviation of sample item, and G represents shrimp gut.

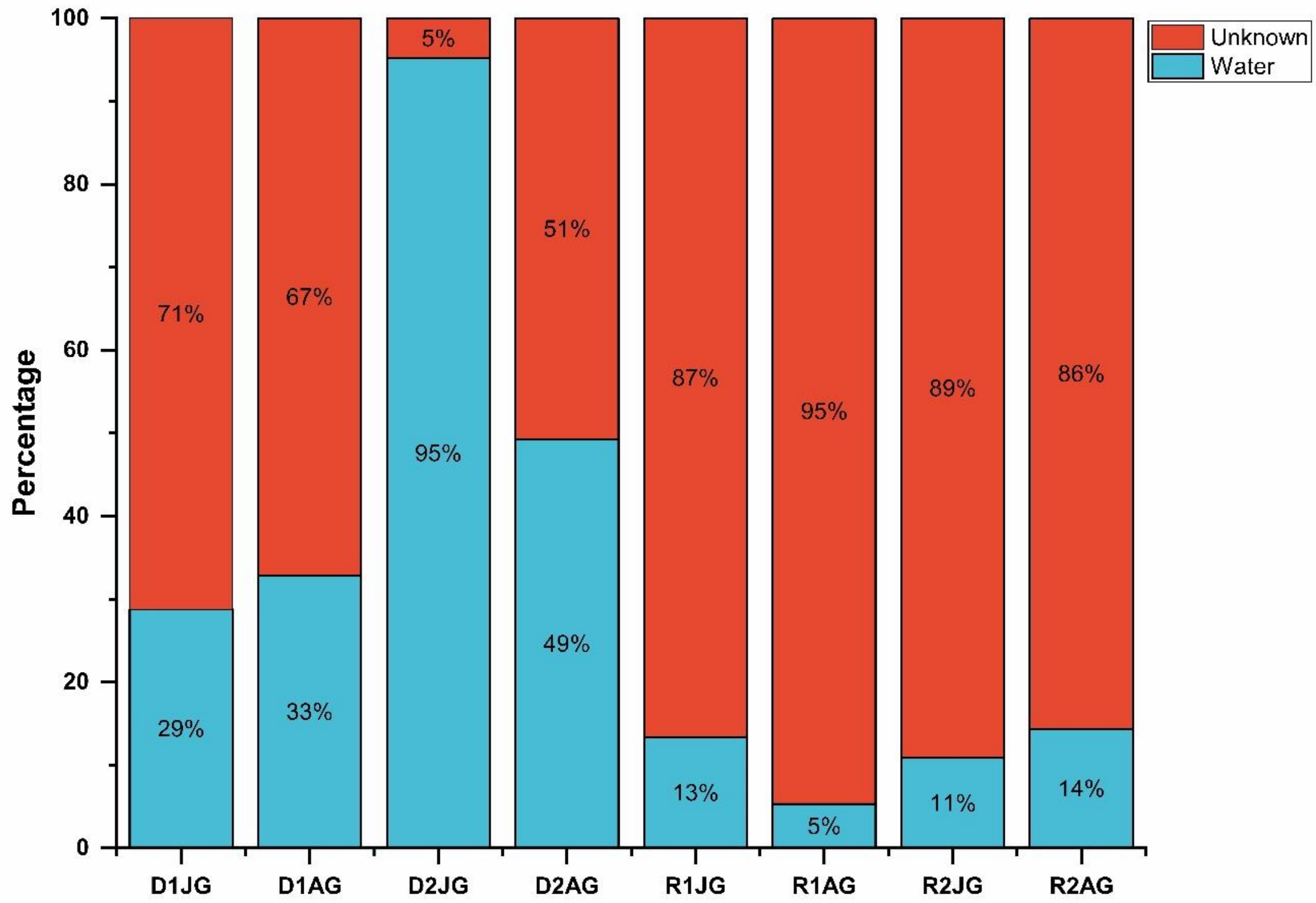

Fig. S7 Source tracking of *L. vannamei* shrimp gut bacterial community estimated within a Bayesian framework in SourceTracker approach. As to the sample names, D1 and D2 represent two farms in Dongying city, R1 and R2 represent two farms in Rizhao city, J and A represent juvenile and adult shrimp, respectively, and G represents gut.

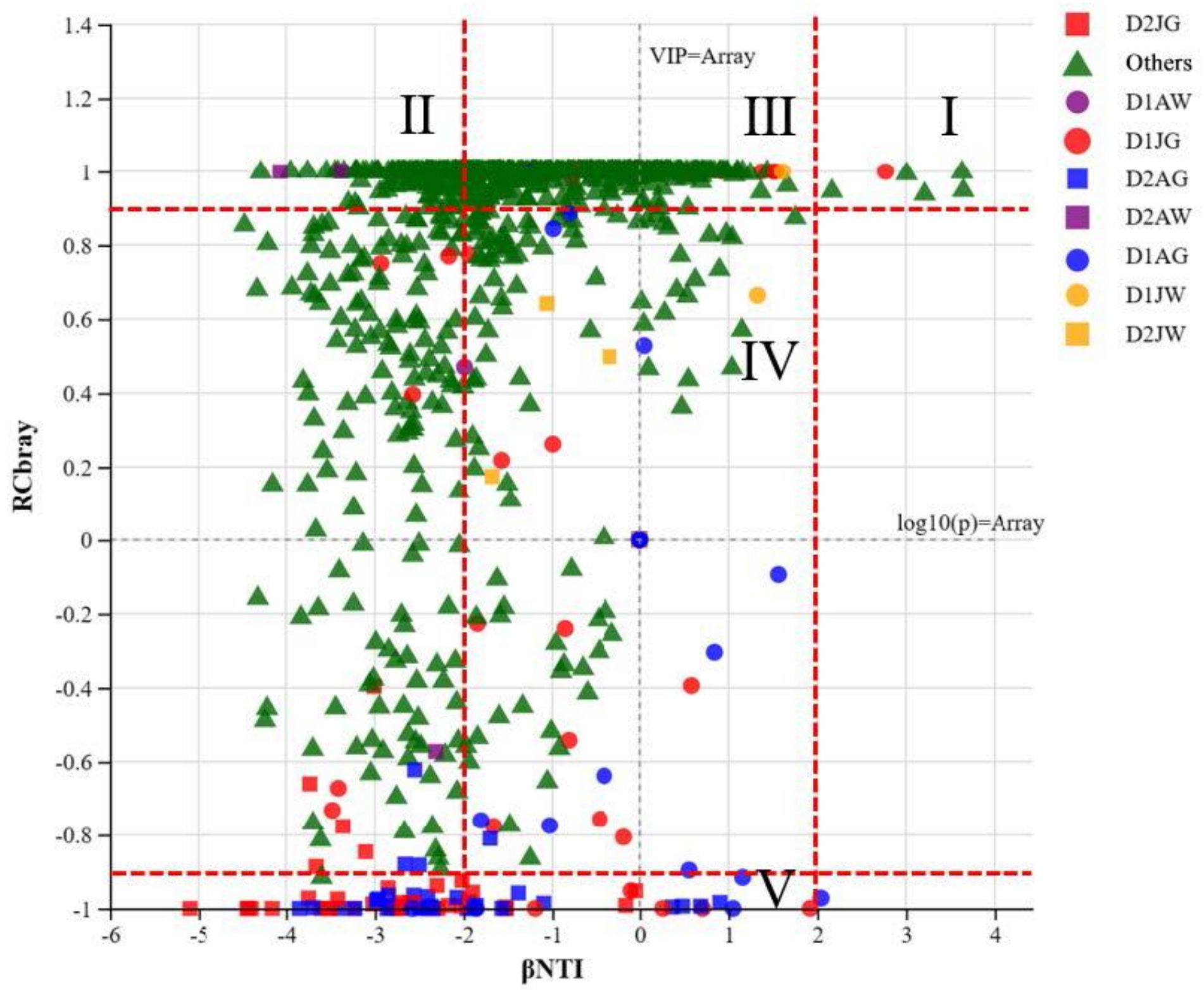

Fig. S8 Structure analysis of bacterial community of *L. vannamei* shrimp and rearing water based on βNTI/RCbray values. As to the sample names, D1 and D2 represent two farms in Dongying city respectively, J and A represent juvenile and adult shrimp respectively, G represents gut, and W represents rearing water.

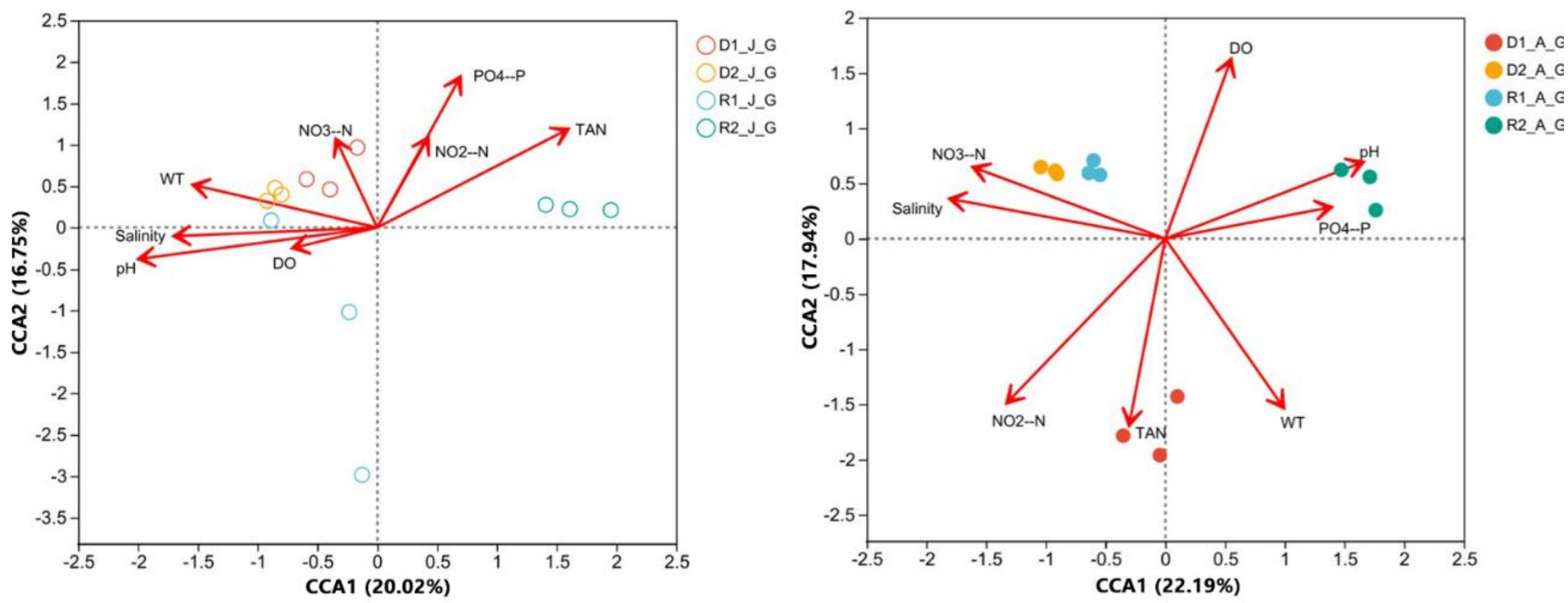

Fig. S9 Canonical correlation analysis (CCA) of the gut bacterial communities and rearing water quality parameters of *L. vannamei* shrimp juvenile (a) and adult (b). As to the sample names, D1 and D2 represent two farms in Dongying city, R1 and R2 represent two farms in Rizhao city, J and A represent juvenile and adult shrimp respectively, G represents gut. In the figure, WT represents water temperature, DO represents dissolved oxygen, NO3-N represents nitrate, NO2-N represents nitrite, TAN represents total ammonia nitrogen, and PO4-P represents phosphate.

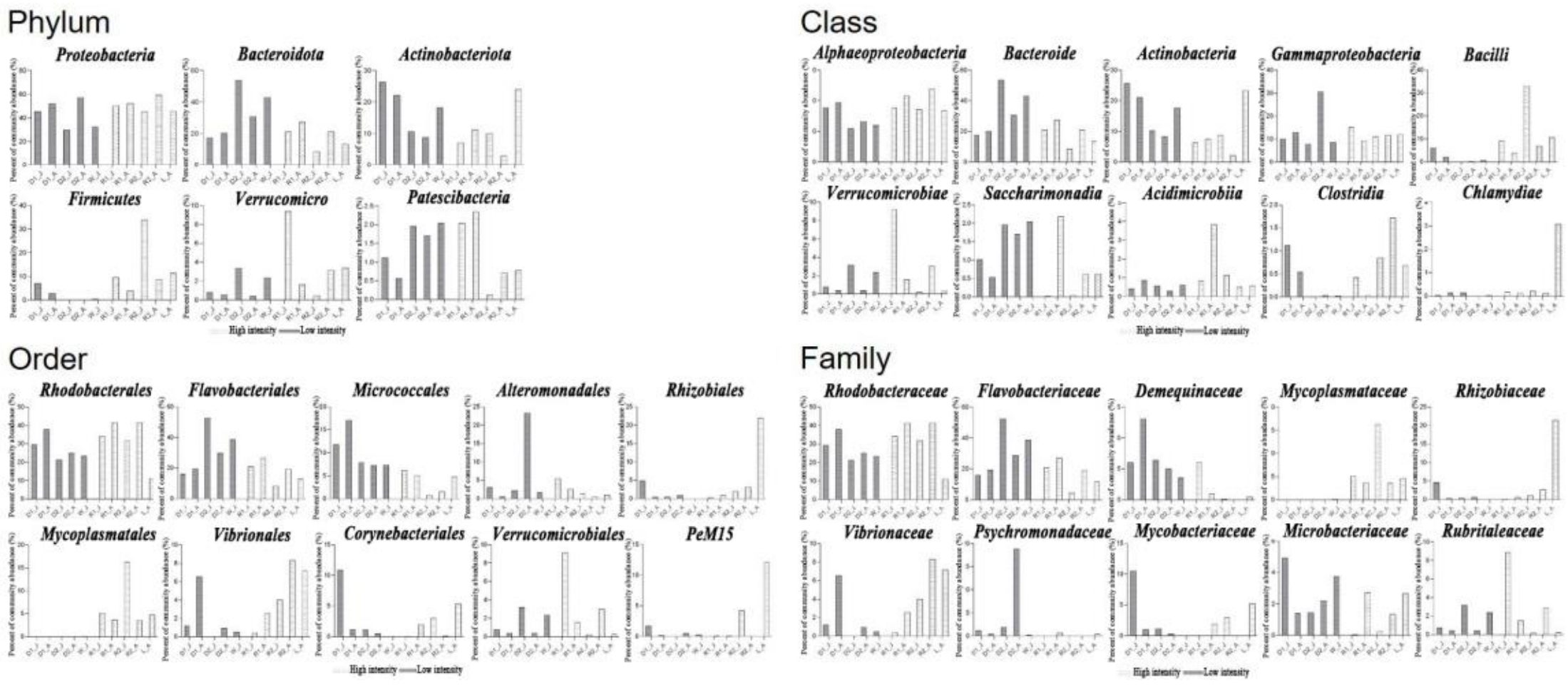

Fig. S10 Comparison of core gut bacterial community of *L. vannamei* shrimp raised under different light intensities (low and high) at phylum, class, order, and family levels respectively. The first character is the abbreviation of farm location, and D represents Dongying city, R represents Rizhao city, W represents Weihai city, and L represents Lianyungang city. The second character is the abbreviation of shrimp stage, and J represents juvenile, while A represents adult. The third character is abbreviation of sample item, and G represents shrimp gut.

Table S1 Body weight and physiochemical conditions of the sampling *L. vannamei* at juvenile and adult stage.

Site	Shrimp body weight (g)		Light intensity/Lux		Temperature/°C		DO/ mg/L		pH		Salinity	
	Juvenile	Adult	Juvenile	Adult	Juvenile	Adult	Juvenile	Adult	Juvenile	Adult	Juvenile	Adult
D1	1.73±0.71	10.63±1.99	830.8±11.5	863.0±15.4	28.97±0.67	29.00±0.10	5.81±0.71	4.70±0.03	7.79±0.11	7.34±0.13	27.96±0.10	27.96±0.02
D2	3.11±0.93	9.39±2.69	848.2±5.2	867.5±14.2	28.90±0.00	26.77±0.32	4.84±0.17	5.52±0.63	8.02±0.01	7.55±0.08	35.98±0.02	33.82±0.06
W	2.49±1.01	NA	886.3±15.6	NA	25.63±0.06	NA	4.84±0.17	NA	8.02±0.01	NA	20.26±0.41	NA
R1	1.32±0.63	8.13±3.05	1034.5±33.1	1286.3±21.0	27.20±0.50	25.00±0.21	5.68±0.12	6.19±0.62	7.90±0.02	7.26±0.07	30.49±0.04	29.35±0.74
R2	0.91±0.44	24.12±5.58	1065.8±38.4	1291.8±12.5	26.53±0.40	28.01±0.15	4.72±0.56	6.14±0.29	7.41±0.05	7.96±0.12	25.07±1.22	26.43±0.04
L	NA	20.27±4.22	NA	1166.5±37.3	NA	28.90±0.00	NA	6.66±0.36	NA	7.30±0.09	NA	35.98±0.02

The meaning of characters of sampling sites are as follows: D: Dongying, Shandong, R: Rizhao, Shandong, W: Weihai, Shandong, L: Lianyungang, Jiangsu.

Table S2 Nutrients concentration of the rearing water collected at juvenile and adult stage *L. vannamei*.

Site	TAN*		Nitrite (mg/L)		Nitrate (mg/L)		Phosphate (mg/L)	
	Juvenile	Adult	Juvenile	Adult	Juvenile	Adult	Juvenile	Adult
D1	16.14±0.33	21.41±1.52	1.60±0.03	1.81±0.05	1.83±0.34	0.47±0.02	0.09±0.01	0.08±0.00
D2	8.90±0.11	13.77±6.83	0.45±0.29	1.34±0.22	0.43±0.20	1.49±0.50	0.08±0.09	0.10±0.06
W	8.06±3.08	NA	1.75±0.07	NA	3.65±0.63	NA	0.21±0.01	NA
R1	5.63±1.70	5.16±1.25	0.36±0.01	0.57±0.06	0.20±0.01	0.69±0.12	0.06±0.01	0.07±0.01
R2	10.78±0.37	8.82±0.05	1.00±0.05	0.07±0.01	0.41±0.11	0.18±0.00	0.09±0.01	0.11±0.01
L	NA	9.73±1.20	NA	1.56±0.07	NA	0.44±0.06	NA	0.04±0.00

* Total ammonia nitrogen.

The meaning of characters of sampling sites are as follows: D: Dongying, Shandong, R: Rizhao, Shandong, W: Weihai, Shandong, L: Lianyungang, Jiangsu
